# The StBBX24 protein affects the floral induction and mediates salt tolerance in *Solanum tuberosum*

**DOI:** 10.3389/fpls.2022.965098

**Published:** 2022-09-07

**Authors:** Agnieszka Kiełbowicz-Matuk, Klaudia Grądzka, Magdalena Biegańska, Urszula Talar, Jagoda Czarnecka, Tadeusz Rorat

**Affiliations:** Department of Regulation of Gene Expression, Institute of Plant Genetics, Polish Academy of Sciences, Poznan, Poland

**Keywords:** antioxidant enzymes, double B-box protein, flowering time, flowering-related genes, salinity, sodium transporters, *Solanum tuberosum*

## Abstract

The transition from vegetative growth to reproductive development is a critical developmental switch in flowering plants to ensure a successful life cycle. However, while the genes controlling flowering are well-known in model plants, they are less well-understood in crops. In this work, we generated potato lines both silenced and overexpressed for the expression of *StBBX24*, a clock-controlled gene encoding a B-box protein located in the cytosol and nuclear chromatin fraction. We revealed that *Solanum tuberosum* lines silenced for *StBBX24* expression displayed much earlier flowering than wild-type plants. Conversely, plants overexpressing *StBBX24* mostly did not produce flower buds other than wild-type plants. In addition, RT-qPCR analyses of transgenic silenced lines revealed substantial modifications in the expression of genes functioning in flowering. Furthermore, *S. tuberosum* lines silenced for *StBBX24* expression displayed susceptibility to high salinity with a lower capacity of the antioxidant system and strongly decreased expression of genes encoding Na^+^ transporters that mediate salt tolerance, contrary to the plants with *StBBX24* overexpression. Altogether, these data reveal that StBBX24 participates in potato flowering repression and is involved in salt stress response.

## Introduction

Proper timing of the transition from vegetative growth to reproductive development is essential for a successful plant life cycle and also has commercial significance for crop plants. Flowering time is determined by the interaction between endogenous genetic components as well as various environmental factors, especially day length and temperature (Ohto et al., 2001; Parcy, 2005; Amasino, 2010; Fornara et al., 2010; Seo et al., 2011; Srikanth and Schmid, 2011; Song et al., 2013). The transition from vegetative to floral development in higher plants is programmed by the simultaneous occurrence of several of these signals and underlies adaptation to different environments (Abelenda et al., 2014). While the mechanisms controlling flowering are well-known in the long-day (LD) plant *Arabidopsis thaliana* and short-day (SD) plant *Oryza sativa*, they are still little known in other plants.

The photoperiod-dependent mechanism promoting flowering in both LD- and SD-type plants is mainly based on the CO (CONSTANS) – FT (FLOWERING LOCUS T) regulon, where the CO protein is an inducer of *FT* expression (Johansson and Staiger, 2015). The transcriptional and post-translational factors regulating *FT* expression vary considerably in response to upstream environmental and endogenous signals among taxa (Ballerini and Kramer, 2011; Abelenda et al., 2014; Johansson and Staiger, 2015). In Arabidopsis, a facultative long-day species, the induction of *FT* expression for flowering during long days depends on the stability of the CO protein, which is regulated by the balanced action of GI (GIGANTEA), FKF1 (FLAVIN BINDING KELCH REPEAT F-BOX PROTEIN 1), and ZTL (ZEITLUPE; Pajoro et al., 2014; Song et al., 2014). The FT florigen protein complexes with FD, a bZIP transcription factor that activates the expression of several *SQUAMOSA PROMOTER-BINDING PROTEIN-LIKE* transcription factors, which in turn induce *FLORAL MERISTEM IDENTITY* genes (Torti and Fornara, 2012).

Meanwhile, the regulation of flowering is more complex in species belonging to *Solanaceae*, such as potato (*Solanum tuberosum*) and tomato (*Solanum lycopersicum*). Floral promotion in these species is regulated by *FT*-like genes that function as inducers or repressors (Molinero-Rosales et al., 2004; Ballerini and Kramer, 2011; Abelenda et al., 2014, 2016). *FT* homologs in tomato and potato constitute a redundant family of transcription regulators that play important roles in flowering time, inflorescence architecture andin other developmental processes. They are divided into three major clades: *FT*-like, *CEN*/*TFL*-like, and *MFT*-like (Hedman et al., 2009). *FT*-like genes promote floral development in contrast to members of the *CEN/TFL* clade, which delay flowering and maintain indeterminacy in inflorescence meristem. *MFT*-like genes are implicated in seed development and germination (Hedman et al., 2009; Nakamura et al., 2011). Furthermore, the role of StBBX1 (StCOL1, previously named StCO) protein in potato flowering and tuberization has previously been reported (González-Schain et al., 2012; Abelenda et al., 2016). Potato StBBX1 protein controls *StSP6A* expression through direct activation of an additional FT family member, *StSP5G*, which in turn acts as a repressor of *StSP6A* transcription in leaves (Abelenda et al., 2016).

The CONSTANS (CO, also termed BBX1) protein belongs to the family of B-box type of zinc finger proteins named BBX. These proteins are transcription regulators forming complex families in plants (Grądzka and Kiełbowicz-Matuk, 2020; Talar and Kiełbowicz-Matuk, 2021), which widely differ in structure and fulfill distinct functions in the regulation of plant growth and development, including seedling photomorphogenesis (Datta et al., 2006, 2007, 2008; Indorf et al., 2007; Chang et al., 2008; Khanna et al., 2009; Holtan et al., 2011; Fan et al., 2012; Gangappa et al., 2013), photoperiodic regulation of flowering (Wenkel et al., 2006; Valverde, 2011), shade avoidance (Crocco et al., 2010) and responses to biotic and abiotic stresses (Nagaoka and Takano, 2003; Wang et al., 2013; Kiełbowicz-Matuk et al., 2014). As shown in Arabidopsis, several BBX proteins, in addition to CO participate in the control of flowering time through CO-dependent or CO-independent pathways. Thus, BBX6 (COL5) stimulates flowering by increasing *CO* expression under SD, whereas BBX7 (COL9) and BBX4 (COL3) suppress flowering by negatively regulating *CO* expression under LD and both LD and SD, respectively (Cheng and Wang, 2005; Datta et al., 2006; Ito et al., 2012; Gangappa and Botto, 2014). In addition, recent studies have revealed that other B-box members lacking the CCT domain regulate flowering *via* distinct overlapping or divergent functions. For instance, in Arabidopsis, overexpression of *BBX24* promotes flowering in both LD and SD, while its deficiency delays flowering only in SD (Li et al., 2014).

We previously identified the *BBX24* gene in two *Solanum* species and showed that its expression exhibited circadian cycling with significant increases in transcript and protein abundances after 8 h of light and a noticeable decrease with minima after 4 h in the darkness. Moreover, we revealed that the *BBX24* gene was induced by high salinity being gated by the time of the day (Kiełbowicz-Matuk et al., 2014). In the present study, we explore the physiological function of StBBX24 in potato (currently called StBBX20, based on the present classification by Talar et al. (2017)) using silenced and overexpressed lines. We show that silenced lines flower earlier than wild-type plants, while lines with overexpression either do not produce flower buds or reveal late flowering. Interestingly, the lack of StBBX24 protein affects the expression pattern of critical genes associated with the floral transition process, unveiling a function of StBBX24 as a repressor of flowering in *Solanum tuberosum*. In other respects, we reveal that silenced lines display decreased tolerance to high salinity with a lower capacity of the antioxidant system and strongly decreased expression of genes encoding Na^+^ transporters, while the plants with overexpression of *StBBX24* display the opposite response.

## Materials and methods

### Plant material, growth conditions and salt treatment

*Solanum tuberosum* L., cv. Desirée plants were propagated *in vitro* on solid MS medium from tuber single-eye plugs at 20/15°C (day/night) under a 150 μmol photon m^−2^ s^−1^ PFD and a 14-h photoperiod for 3 weeks. Four single-eye plugs from tubers were transferred on soil in plastic pots (diameter 21 cm) in the phytotron and grown under standard conditions (20 ± 1°C, 40% relative humidity and PFD of 350 μmol photons m^−2^ s^−1^) under 16-h-light/8-h-dark or 14-h-light/10-h-dark conditions. In these conditions, the term Zeitgeber time (ZT) refers to the experimental time (Johnson et al., 2003; Beneragama and Goto, 2010), and the ZT0 point corresponds to light on (initiation of experimental dawn). Two independent experiments and two independent plant samples from each experiment were used. The times of the first visible flower buds and the first flower opening were recorded. Tubers from twenty plants per genotype (four plants per plastic pot) were analyzed. The potato tubers were placed on a flat surface. The three mutually perpendicular axes of the potato tubers were designated and measured using a vernier caliper. The longest point of intersection depicted the length of the tuber, the longest intersection point perpendicular to the length represented the width of the tuber, and the longest intersection point perpendicular to the width and length represented the thickness of the tuber. The shape index of the measured tubers was calculated according to Ismail (1988).

For salt exposure, 3-week-old phytotron-grown plants were irrigated with an equal amount (per pot) of 0.15 M NaCl solution (−0.99 MPa osmotic potential) for 8 days. Plant material was harvested 8 h, 3 and 8 days after salt application.

### Biomass determination

The biomass of aerial parts was determined for plants grown under normal conditions or subjected to high salt. The samples were collected at ZT6, and excised shoots were dried at 80°C overnight before weighing. Sixteen plants per genotype and growth condition were analyzed.

### Analysis of chlorophyll content

One-cm diameter disks were excised from well-expanded leaves of plants grown under control conditions or subjected to high salt, then crushed in 2 ml 80% acetone. After storing 1 h in the dark at 4°C and centrifugation (8,000 *g*, 10 min), the content in chlorophylls *a* and *b* was measured spectrophotometrically and calculated according to (Lichtenthaler, 1987).

### The activity of antioxidant enzymes

The total activity of Superoxide Dismutase and Peroxidase was assayed using the Superoxide Dismutase Activity Kit (MAK379, Merck) and Peroxidase Activity Assay Kit (MAK092, Merck), respectively, according to the protocols. Homogenates were prepared from pooled leaves (100 mg) in 0.1 M Tris–HCl, pH 7.4 containing 0.5% Triton X-100, 5 mM β-mercaptoethanol and 1 mM PMSF, incubated at 4°C for 30 min, and centrifuged at 14,000 *g* for 10 min. One Unit of SOD activity was defined as the amount of enzyme that inhibits the xanthine oxidase activity at 37°C. One unit of peroxidase activity was defined as the amount of enzyme that reduces 1.0 μmole of H_2_O_2_ per minute at 37°C. The soluble protein content was determined for all samples to normalize each enzyme activity between different trials. The activity measurements were performed spectrophotometrically at 410 nm and 570 nm wavelengths for SOD and peroxidase, respectively.

### Quantitative real-time PCR analysis of gene expression

Total RNA was prepared from potato leaves and apical shoot parts using “TRI Reagent RT” (Molecular Research Center, Inc.) according to the manufacturer’s protocol. Equal RNA amounts were treated with RNase-free DNaseI (Ambion). The RNA concentration was measured using a Nano Drop photometer (Thermo Scientific). 2 μg of purified RNA was used for cDNA synthesis. The first DNA strand was prepared using the Thermo Scientific Maxima Reverse Transcriptase. Real-time qPCR was performed using a CFX96 Touch™Real-Time PCR detection system (Bio-Rad, Hercules, United States) and Maxima SYBR Green/ROX qPCR Master Mix (Thermo). Each assay using gene-specific primers (Supplementary Table S1) amplified a single product of the appropriate size with high PCR efficiency (90%–110%). The fluorescence data analysis was conducted using the CFX™ Software (Version 3.0, Bio-Rad, Hercules, United States). All RT-qPCR analyses were normalized using the threshold cycle (*C*_1_) values corresponding to the *EF-1-α* and/or the *18S* reference genes (Nicot et al., 2005; Kiełbowicz-Matuk et al., 2014). The normalized expression of the target gene (ΔΔCq) was calculated as the relative quantity of target normalized to the quantities of the two reference genes, according to the manufacturer’s software. The values presented are the means of two technical replicates from two independent biological samples.

### Protein extracts preparation

Nuclei-enriched fractions were isolated from 3-week-old *S. tuberosum* leaves at ZT6 as described by Kiełbowicz-Matuk et al. (2017). Further purification of nuclear proteins was performed by suspending the nuclear pellet in two volumes of 25 mM Tris–HCl, pH 8.0, 30 mM NaCl, 2 mM EDTA, and 1% Nonidet P-40 supplemented with Halt Protease and Phosphatase Inhibitor Cocktail (Thermo Scientific) and incubation at 4°C for 30 min. Samples were then centrifuged at 13,000 *g* for 10 min to pellet the insoluble chromatin and collect the supernatant (nucleoplasm fraction). The chromatin pellet was washed and resuspended in 50 mM Tris–HCl, pH 8.0, 30 mM NaCl, 1 mM PMSF, 1% sodium deoxycholate, 1% SDS, 0.5% Triton X-100, and incubated at 4°C for 1 h before centrifugation at 14,000 *g* for 10 min. Proteins from the nucleoplasm and chromatin fractions were precipitated with four volumes of acetone.

Cytosolic proteins were prepared from young leaves of 3-week-old plants at ZT6 in a buffer containing 0.3 M sucrose, 50 mM Tris–HCl, pH 8.0, 10 mM MgCl_2_, 1 mM EDTA, and 1 mM PMSF, as described by Kiełbowicz-Matuk et al. (2014). The homogenate was filtered through a two-layer Miracloth (Calbiochem) and centrifuged at 3,000 *g* for 10 min at 4°C. Then, the cleared supernatant was centrifuged at 20,000 *g* for 20 min at 4°C and collected. Cytosolic proteins were precipitated with four volumes of acetone. Soluble proteins were extracted from young leaves of 3-week-old plants at ZT6 in a buffer containing 50 mM Tris–HCl, pH 8.0, 2% β-mercaptoethanol, and 1 mM PMSF.

### Western blot analysis

Protein concentration was measured using a modified Lowry copper-based method (Pierce). Proteins were separated in SDS-PAGE gels containing 13% acrylamide, and then electroblotted onto a 0.22 μm nitrocellulose membrane (Schleicher and Schuell, Germany) using a semi-dry blotting apparatus (Bio-Rad). Membranes were stained with Ponceau red to ensure that equal protein amounts were transferred in each lane. The Western blot analysis was performed using the StBBX24 antiserum diluted 1:500. Sera raised against nuclear cap-binding protein subunit 2 (CBP20), histone H3, and UDP-glucose pyrophosphorylase (UGPase) were purchased from Agrisera (Vännäs, Sweden) and used diluted 1:500, 1:5,000, and 1:1,000, respectively. Sera raised against cytosolic cyclophilin (*Ss*CyP) was diluted 1:500 (Kiełbowicz-Matuk et al., 2007). As a loading control to directly detect the total protein concentration for cytosolic fraction, antibodies raised against cytosolic marker UGPase protein were used. Anti-histone H3 antibodies were used as a loading control for nuclear proteins (Kiełbowicz-Matuk et al., 2017). Bound antibodies were detected using an anti-rabbit immunoglobulin G (horseradish peroxidase conjugate) diluted 1:10,000 (Roche) and a chemiluminescent substrate (Western Bright Quantum, Advansta). The Western blotting was performed using a G:BOX Imaging System (Syngene). Band intensity was analyzed using GeneSys software V1 6.5.0 (Syngene). Analyses were performed using protein extracts from two independent sets of plants per condition, and at least three replicates were conducted for each condition. Mean values ± SD from two biological and three technical replicates are presented.

### MicroRNA targeting and vector construction for *StBBX24* overexpression

To investigate the physiological function of StBBX24 in *Solanum tuberosum* plants, we designed artificial microRNAs (amiRNAs) targeting the *StBBX24* mRNA to generate transgenic gene-silenced lines. Because cultivated potato species are autotetraploid and highly heterozygous, we sequenced multiple *StBBX24* cDNA clones from the Desirée cultivar. *StBBX24* mRNA fragments showing no allelic polymorphism were selected and analyzed to find the most effective target sites for RNA-induced silencing complex (RISC) slicing using Web MicroRNA Designer.^1^ Four *StBBX24* allele variants were identified (Supplementary Figures S1A,B). The mRNA target sites within *StBBX24* cDNA are marked in Supplementary Figure S1. Using the same platform, three amiRs were selected (Supplementary Table S1). Post-transcriptional silencing using artificial miRNA was performed as in Schwab et al. (2006) and Ossowski et al. (2008). Briefly, a set of overlapping PCRs was performed using the appropriate primers for the three constructs (Supplementary Table S1) to exchange miR319b/miR319b* for amiRBBX24.1/amiRBBX24.1*, amiRBBX24.2/amiRBBX24.2*, and amiRBBX24.3/amiRBBX24.3*. The DNA fragments carrying *aMIRBBX24.1*, *aMIRBBX24.2*, and *aMIRBBX24.3* genes were cloned into the pHannibal vector (AJ311872; Wesley et al., 2001). The DNA constructs, including the CaMV 35S promoter and an OCS terminator, were transferred into a pART27 vector (Gleave, 1992). As a result, 92 transgenic lines harboring the T-DNAs containing the *aMIRBBX24.1*, *aMIRBBX24.2* or *aMIRBBX24.3* genes were generated. All lines were assayed for silencing efficiency using RT-qPCR (Supplementary Figure S2A). Forty lines exhibiting strong silencing of *StBBX24* expression were chosen for the Western blot analysis. Among them, 10 lines harboring the *aMIRBBX24.1* gene, i.e., amiRBBX24.1.1, amiRBBX24.1.5, amiRBBX24.1.9, amiRBBX24.1.10, amiRBBX24.1.12, amiRBBX24.1.13, amiRBBX24.1.15, amiRBBX24.1.17, amiRBBX24.1.25, and amiRBBX24.1.26, showed complete silencing at the protein level (Supplementary Figure S2B).

The *StBBX24* overexpression lines (*StBBX24*-OE) were generated by recombining the *StBBX24* full-length coding sequence from the pENTR clone with the pGWB402 binary vector *via* the Gateway LR Clonase system (Invitrogen). To generate the pENTR™/SD/D-TOPO^®^ vector cDNA for *StBBX24* was amplified using Phusion™ High-Fidelity DNA polymerase (Thermo Scientific) and gene-specific primer pairs (Supplementary Table S1), cloned into pENTR and then sequenced. pGWB402 was a gift from Tsuyoshi Nakagawa (Addgene plasmid #74796, http://n2t.net/addgene:74796;RRID:Addgene_74796; Nakagawa et al., 2007).

The plasmids were separately introduced into *Agrobacterium tumefaciens* strain LBA4404 and transformed into *S. tuberosum* by the *Agrobacterium*-mediated technique (Chen et al., 1994). Plants were regenerated as described by Mac et al. (2004).

Total genomic DNA and cellular RNA were isolated from potato leaves using a GeneJET Plant Genomic DNA Purification Kit (Thermo Scientific) and TRI Reagent RT (Molecular Research Center, Inc.), respectively. Transformed lines were analyzed using PCR and primers specific to the transgene. In addition, *StBBX24* expression was assayed using quantitative real-time PCR and Western blot analysis, as described previously.

## Results

### Abundance of *StBBX24* in apical organs

**Figure 1:**
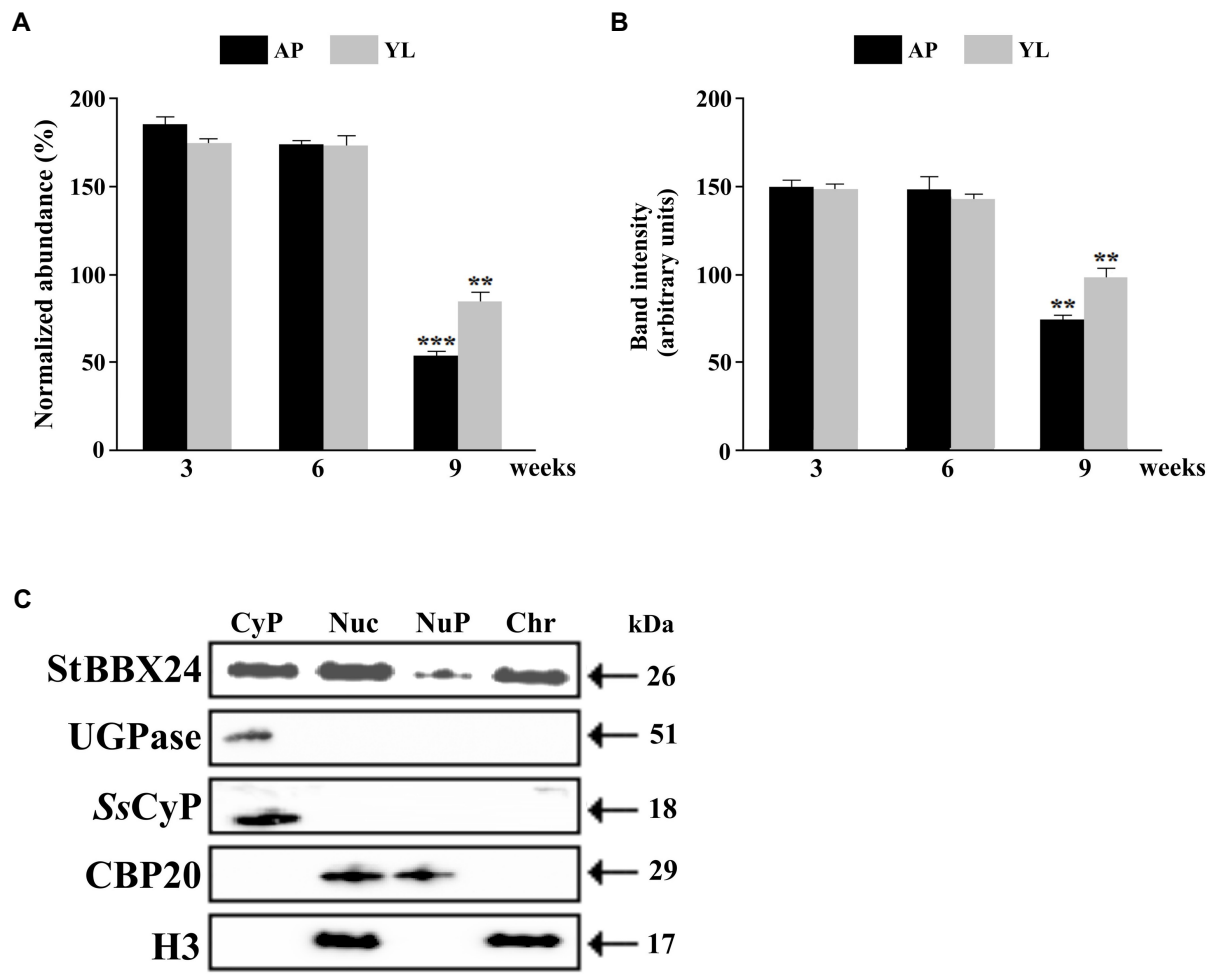
Expression of *StBBX24* in young organs of *S. tuberosum* plants and nuclear localization of StBBX24. StBBX24 transcript **(A)** and protein **(B)** abundance in apical shoot parts and young leaves of phytotron-grown *S. tuberosum* plants. **(A)** Quantitative RT-PCR was performed as described in Materials and methods. **(B)** Western analysis of StBBX24 abundance was performed using sera raised against StBBX24 as described in Materials and methods. As a loading control of the total protein for cytosolic fraction, antibodies raised against cytosolic marker UGPase protein were used. AP – apical shoot part; YL – young leaves (including very young developing leaves). **(C)** Western blot analysis of proteins from cytosolic (Cyt), total nuclei (Nuc), nucleoplasm (NuP) and chromatin (Chr) fractions prepared from leaves of *S. tuberosum* plants. 10–40 μg proteins from nuclei-enriched and cytosolic fractions were set per lane. The analysis was carried out using sera raised against StBBX24, UDP-glucosepyrophosphorylase (UGPase), cytosolic cyclophilin (CyP), histone H3 and cap-binding protein subunit 2 (CBP20). Means ± SD of four values from two independent experiments (two replicates per experiment) are presented. Statistical analysis was performed using the *t-*test (Statistica program, version 10). ** and ***, values significantly different from the 3-week old plants value with *p* < 0.01 and *p* < 0.001, respectively.

We previously reported that *StBBX24* gene expression, was differentially regulated as a function of organ type during vegetative growth and reproductive development (Kiełbowicz-Matuk et al., 2014). In the present study, we further analyzed *StBBX24* expression in young leaves (<2 cm in length) and apical shoot parts of 3-, 6-, and 9-week-old *S. tuberosum* plants grown under a 16 h photoperiod. At these stages, the stem length considerably varied (10, 20 and 35 cm at 3, 6, and 9 weeks, respectively) as well as the number of well-expanded composed leaves (0, 10, and 15, respectively). Most of the 9-week-old *S. tuberosum* plants exhibited emerged, but non open, flower buds. As shown in Figures 1A,B, similar *StBBX24* transcript and protein amounts were observed in extracts from young leaves and apical shoot parts of 3- and 6-week-old plants. In comparison, the transcript and protein abundances in 9-week-old plants were much lower, two- to three-fold less, particularly in apical parts, revealing that the *StBBX24* expression changes during the plant growth with a decreases in developing of floral organs.

### Sublocalization of StBBX24

We have previously shown that the StBBX24 protein is presented both in the nucleus and cytosol, and its distribution depends on the time of day (Kiełbowicz-Matuk et al., 2014). Here, we investigated its subnuclear localization in nucleoplasm and chromatin fractions from leaves of 3-week-old *S. tuberosum* plants at ZT6 when the amount of StBBX24 is the highest in nucleus (Kiełbowicz-Matuk et al., 2014). To validate the fraction purity, a Western blot analysis of proteins restricted to nucleoplasm or chromatin, i.e., cap-binding protein CBP20 and histone H3, respectively, was performed (Agrisera). As shown in Figure 1C, histone H3 was present in the chromatin fraction, whereas CBP20 was localized in the nucleoplasm fraction. Interestingly, the serum raised against recombinant StBBX24 specifically detected the protein in both chromatin and nucleoplasm fractions. However, the band intensity was higher in chromatin than in nucleoplasm, clearly showing that StBBX24 was mainly localized in the chromatin within nucleus.

### Early flowering phenotype of *StBBX24*-silenced potato plants

**Figure 2:**
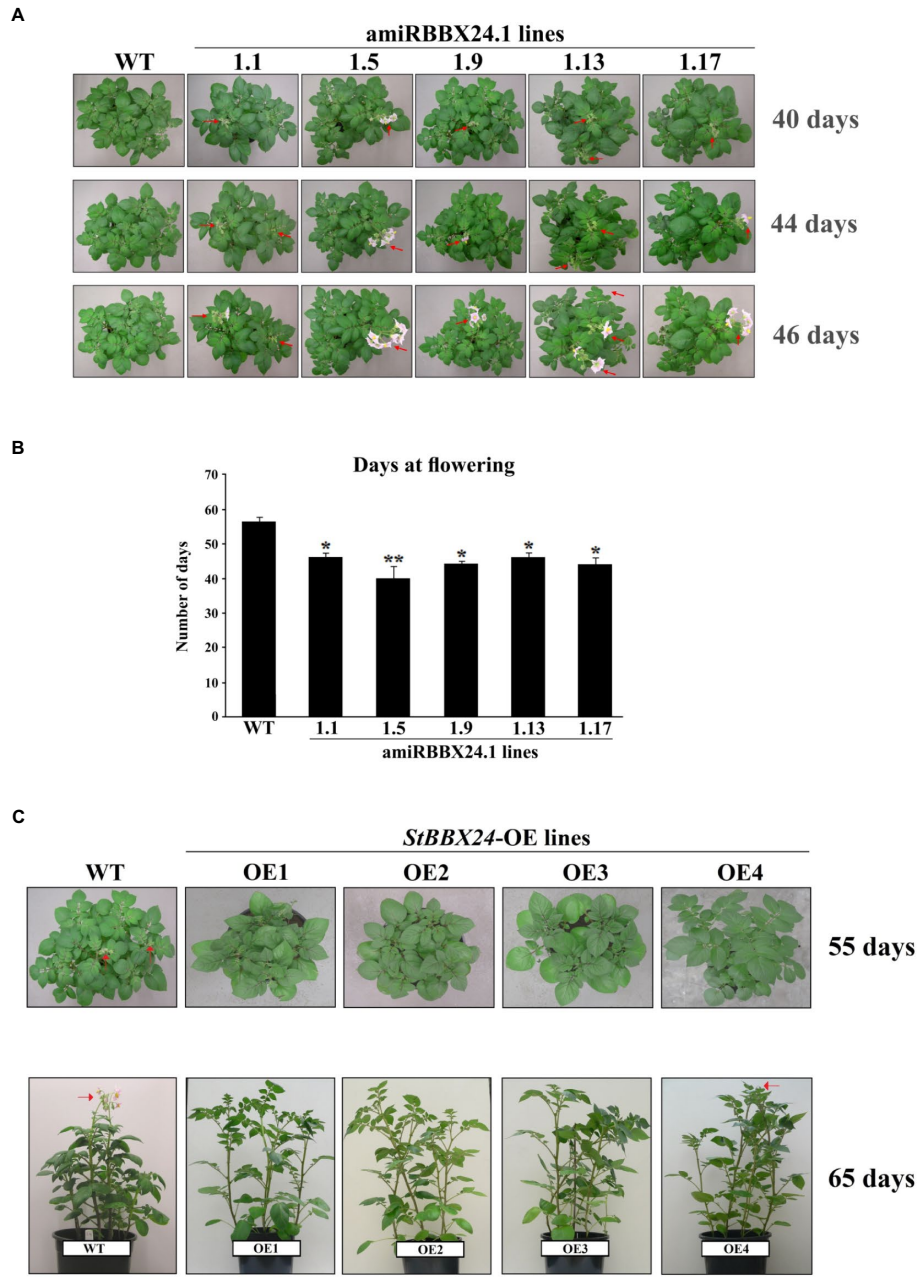
Flowering phenotype of *S. tuberosum* plants silenced for *StBBX24* expression and overexpressing *StBBX24*. **(A)** Phenotype of WT, amiRBBX24.1.1, amiRBBX24.1.5, amiRBBX24.1.9, amiRBBX24.1.13 and amiRBBX24.1.17 transgenic plants at the pre-flowering and flowering stages. Sixteen to twenty plugs from potato tubers per genotype were grown in plastic pots (diameter 21 cm) in the growth room in standard conditions under a 16-h photoperiod. The red arrows indicate the presence of flower buds and open flowers. **(B)** Number of days at flowering in WT and lines silenced for *StBBX24* expression. * and **, values significantly different from the WT value (*t*-test) with *p* < 0.05 and *p* < 0.01, respectively. WT, wild-type. **(C)** Phenotype of WT, *StBBX24*-OE1, *StBBX24*-OE2, *StBBX24*-OE3 and *StBBX24*-OE4 transgenic plants at the pre-flowering and flowering stages. Twenty plugs from potato tubers per genotype were grown in plastic pots (diameter 21 cm) in the growth room in standard conditions under a 16-h photoperiod. The red arrows indicate the presence of flower buds and open flowers.

To gain insight into the role of StBBX24 in cultivated potato development, we studied five silenced lines (amiRBBX24.1.1, amiRBBX24.1.5, amiRBBX24.1.9, amiRBBX24.1.13, and amiRBBX24.1.17) and wild-type plants grown under a 16-h photoperiod. We did not observe any difference concerning the plant architecture, but we did notice slight changes in the number and size of tubers in transgenic lines as compared with wild-type plants (Supplementary Figures S3A,B; Supplementary Tables S2, S3). However, most significantly, a pronounced phenotype was observed regarding flowering time in transgenic lines. Indeed, we noticed that the lack of StBBX24 protein strongly promoted flowering, with the opening of flower buds occurring ~16, 12, and 10 days earlier in amiRBBX24.1.5, amiRBBX24.1.9, amiRBBX24.1.17, amiRBBX24.1.1, and amiRBBX24.1.13 plants, respectively, as compared with in WT (Figures 2A,B). Meanwhile, when potato plants were grown under a shorter photoperiod (12 h), conditions preventing the flower bud differentiation process, no difference was noticed between WT and transgenic lines that both exhibited only vegetative development (Supplementary Figure S3C). These results reveal that StBBX24 may function as a regulator affecting the control of reproductive development in potatoes, likely through a role at the flower bud initiation stage.

### The flowering phenotype of transgenic potato plants overexpressing *StBBX24*

Then we investigated the reproductive development of four *StBBX24* overexpressing potato lines (*StBBX24*-OE1, *StBBX24*-OE2, *StBBX24*-OE3, and *StBBX24*-OE4) and the wild-type control grown under a 16-h photoperiod (Figure 2C). All transgenic lines were assayed for *StBBX24* expression level using Western blot (Supplementary Figure S4). We did not notice any substantial alterations in plant architecture and size of tubers, and the number of tubers, between transgenic plants and their wild type (Supplementary Figures S5A,B; Supplementary Tables S2, S3.). Meanwhile, significant differences in phenotype were observed concerning flowering time in the overexpressing lines compared to the wild-type. We found that the *StBBX24*-OE1, *StBBX24*-OE2, and *StBBX24*-OE3 lines did not produce flower buds. Meanwhile, in the *StBBX24*-OE4 line, only a few flower buds occurred ~10 days later than in WT (Figure 2C). Our data provide evidence that StBBX24 protein may affect the success of potato reproduction.

### Expression of flowering-related genes in *StBBX24*-silenced and overexpressed potato lines

**Figure 3:**
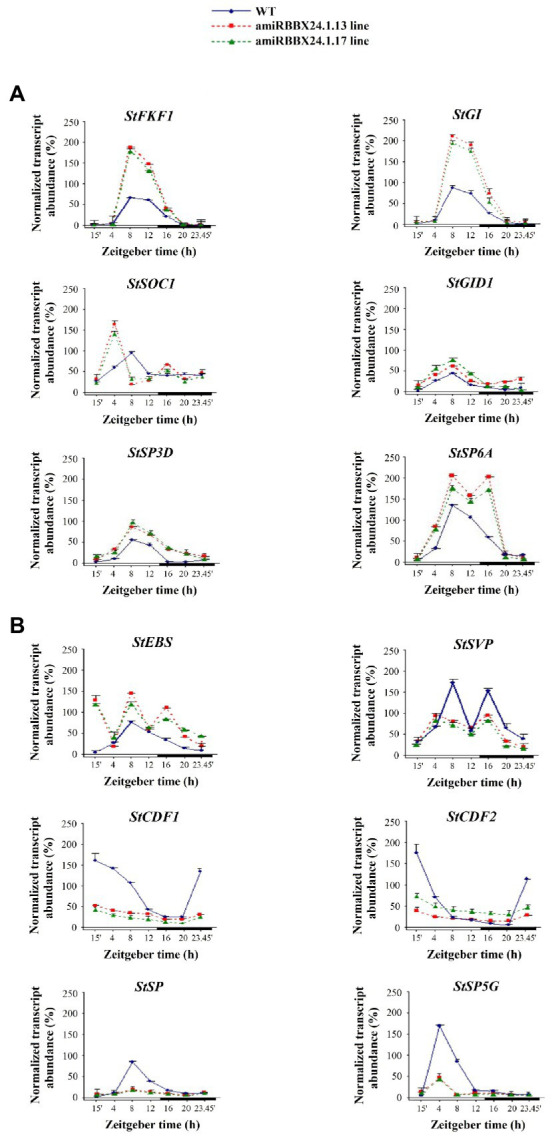
Expression profiles of flowering-related genes in *S. tuberosum* plants silenced for *StBBX24* expression. Quantitative RT-PCR analysis of *StFKF1* (ID: PGSC0003DMG400019971), *StGI* (ID: PGSC0003DMG400018791), *StSOC1* (ID: PGSC0003DMG401024252), *StGID1* (ID: PGSC0003DMG400000139), *StSP3D* (ID: PGSC0003DMG400016179), *StSP6A* (ID: PGSC0003DMG400023365) **(A)** and *StEBS*, *StSVP* (ID: PGSC0003DMG400016203), *StCDF1* (ID: PGSC0003DMT400047370), *StCDF2* (ID: PGSC0003DMT400003359), *StSP* (ID: PGSC0003DMG400007111), *StSP5G* (ID: PGSC0003DMG400023365) **(B)** transcript levels in leaves of WT, amiRBBX24.1.13 and amiRBBX24.1.17 3-week old plants under a 14-h photoperiod at different time points (15′, 4, 8, 12, 16, 20, 23h45′) for 24 h during the light and dark phases. Zeitgeber time (ZT) refers to experimental time where the ZT0 point corresponds to light-on (initiation of experimental dawn). The bars represent the subjective light and night conditions. RT-qPCR analyses were normalized using the threshold cycle (*C*_1_) values corresponding to the *18S* and *EF-1-α* reference genes. The normalized expression of the target gene (ΔΔCq) was calculated as the relative quantity of target normalized to the quantities of the two reference genes according to the manufacturer’s software. Means ± SD of four values from two independent experiments (two replicates per experiment) are presented.

**Figure 4:**
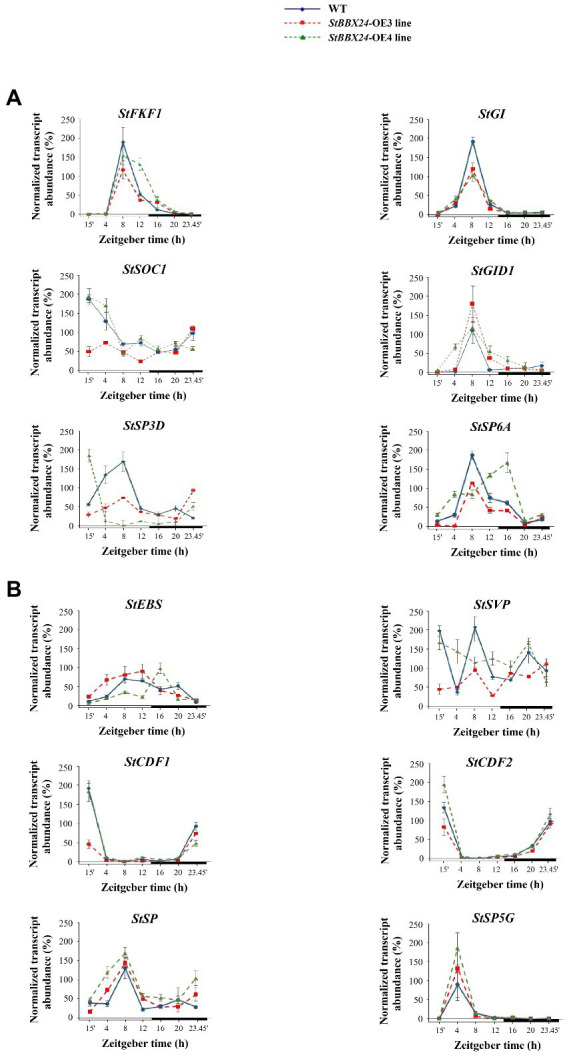
Expression profiles of flowering-related genes in *S. tuberosum* plants overexpressing *StBBX24*. Quantitative RT-PCR analysis of *StFKF1* (ID: PGSC0003DMG400019971), *StGI* (ID: PGSC0003DMG400018791), *StSOC1* (ID: PGSC0003DMG401024252), *StGID1* (ID: PGSC0003DMG400000139), *StSP3D* (ID: PGSC0003DMG400016179), *StSP6A* (ID: PGSC0003DMG400023365) **(A)** and *StEBS*, *StSVP* (ID: PGSC0003DMG400016203), *StCDF1* (ID: PGSC0003DMT400047370), *StCDF2* (ID: PGSC0003DMT400003359), *StSP* (ID: PGSC0003DMG400007111), *StSP5G* (ID: PGSC0003DMG400023365) **(B)** transcript levels in leaves of WT, *StBBX24*-OE3 and *StBBX24*-OE4 3-week old plants under a 14-h photoperiod at different time points (15′, 4, 8, 12, 16, 20, 23h45′) for 24 h during the light and dark phases. The bars represent the subjective light and night conditions. RT-qPCR analyses were normalized using the threshold cycle (*C*_1_) values corresponding to the *EF-1-α* reference genes. The normalized expression of the target gene (ΔΔCq) was calculated as the relative quantity of target normalized to the quantities of the two reference genes according to the manufacturer’s software. Means ± SD of four values from two independent experiments (two replicates per experiment) are presented. Zeitgeber time (ZT) refers to experimental time where the ZT0 point corresponds to light-on (initiation of experimental dawn).

Based on the accelerated flowering of silenced lines and lack or deleted flowering of overexpressed lines, we paid more attention to genes involved in regulating flowering time. We studied the expression patterns as a function of the photoperiod of the key flowering-related genes in amiRBBX24.1.13 and amiRBBX24.1.17 and *StBBX24*-OE3 and *StBBX24*-OE4 lines using RT-qPCR. In the lines with silenced *StBBX24* gene expression, we observed significantly increased expression levels of floral activators, including *StFKF1* (*Flavin-binding, Kelch repeat*, and *F box 1*), *StGI* (*GIGANTEA*), *StGID* (*Gibberellin Insensitive Dwarf 1*), and *StSOC1* (*Suppressor of Overexpression of CO1*). As shown in Figure 3A, the transcript levels of *StFKF1*, *StGI*, *StSOC1*, and *StGID* were considerably higher than in WT. Of note, the expression patterns of these genes were still periodic, but the periods were shifted to the early light phase for *StSOC1* and *StGID1*, as compared with those in WT (Figure 3A). Additionally, we analyzed the expression of critical floral repressors, such as *StEBS* (*Early Bolting in Short days*), *StSVP*, *StCDF1*, and *StCDF2* (*CYCLING DOF FACTORS 1* and *2*), and beyond *StEBS*, we observed a significant decrease in their transcript abundance in silenced lines as compared with WT (Figure 3B). Regarding *StEBS*, the transcript abundance was considerably higher in both silenced lines, but the oscillation pattern was significantly affected (Figure 3B). On the contrary, in *StBBX24* overexpressing lines, we noticed a decrease in the transcript level of some genes encoding flowering activators, including *StFKF1* and *StGI*; Meanwhile, transcript amount for some repressors, such as *StEBS* was increased concerning the wild-type (Figures 4A,B). Notably, the *StSOC* and *StSVP*, displayed impaired expression patterns in both the light and dark phases of the 24-h diurnal cycle, as compared with those in WT (Figures 4A,B).

In *Solanaceae*, the participation of *FLOWERING TIME*-like genes, including *StSP6A* (*SELF PRUNING 6A*) and *StSP3D* (*SELF PRUNING 3D*) as inducers and *StSP5G* (*SELF PRUNING 5G*) and *StSP* (*SELF PRUNING*) as repressors of floral development has been reported. Thus, we investigated their expression in leaves of *StBBX24-*silenced plants and observed that *StSP6A* and *StSP3D* transcript levels were substantially up-regulated as compared with WT (Figure 3A), while those of *StSP5G* and *StSP* were significantly decreased (Figure 3B). Significant changes in transcript accumulation were also observed in plants with *StBBX24* overexpression compared with non-transformed plants (Figures 4A,B). We noted that transcript levels of *StSP6A* and *StSP3D* were substantially down-regulated; in the meantime, *StSP* and *StSP5G* were elevated as compared with WT. Furthermore, it was noted that while the regular expression of *StSP* and *StSP5G* genes was intact, it was disrupted for *StSP3D* and *StSP6A* genes (Figures 4A,B). These findings reveal that the StBBX24 protein is an important regulator of flowering time in cultivated potato.

### Salt stress tolerance of *StBBX24-*silenced and -overexpressed *Solanum tuberosum* plants

As *StBBX24* encodes a protein participating in salt stress responses in *S. tuberosum* and sodium chloride (0.15 and 0.2 M) affect the circadian-regulated amount of BBX24 protein (Kiełbowicz-Matuk et al., 2014), we assessed the influence of StBBX24 on salt stress tolerance in potato by characterising the phenotype of three silenced lines and three overexpressed lines in high salinity conditions. Initially, we performed a phenotypic characterization of *StBBX24-*silenced and -overexpressed representative lines during the vegetative development under normal growth conditions, i.e., amiRBBX24.1.1, amiRBBX24.1.13, amiRBBX24.1.17, and *StBBX24*-OE2, *StBBX24*-OE3, and *StBBX24*-OE4 to evaluate their growth and morphological divergences. No significant difference was noticed in the rate of vegetative growth, the number of nodes and the number, shape and size of leaves (Supplementary Figures S3A, S5A).

**Figure 5:**
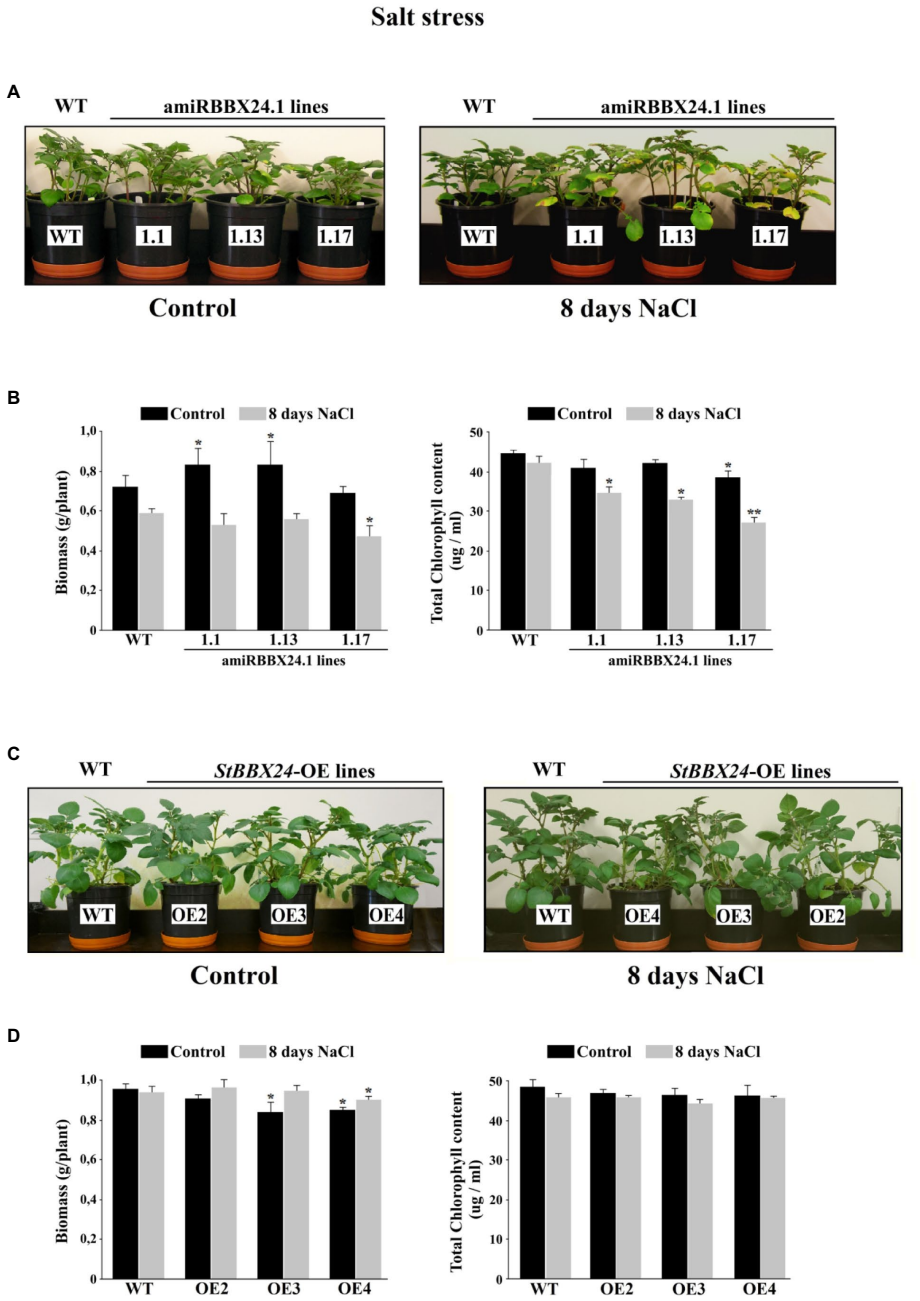
Phenotypic characterization of the *StBBX24*-silenced and -overexpressing *S. tuberosum* lines in response to salinity. **(A,C)** Phenotype of phytotron-grown *S. tuberosum* WT, amiRBBX24.1.1, amiRBBX24.1.13 and amiRBBX24.1.17 plants, and *StBBX24*-OE2, *StBBX24*-OE3 and *StBBX24*-OE4 lines in high salt conditions (0.15 M NaCl). Plugs from potato tubers were grown for 3-weeks in plastic pots in control conditions in a phytotron and were then watered with a solution containing 0.15 M NaCl in the same conditions for 8 days. **(B,D)** Biomass of aerial parts and leaf chlorophyll content of plants grown in control conditions or subjected to high salt for 8 days. Leaf biomass values are mean ± SD from 16 independent measurements per genotype. The chlorophyll content was measured using three 1-cm leaf disks from young well-expanded leaves. Statistical analysis was performed using the *t*-test (Statistica program, version 10). * and **, values significantly different from the WT value with *p* < 0.05 and *p* < 0.01, respectively. WT, wild-type.

**Figure 6:**
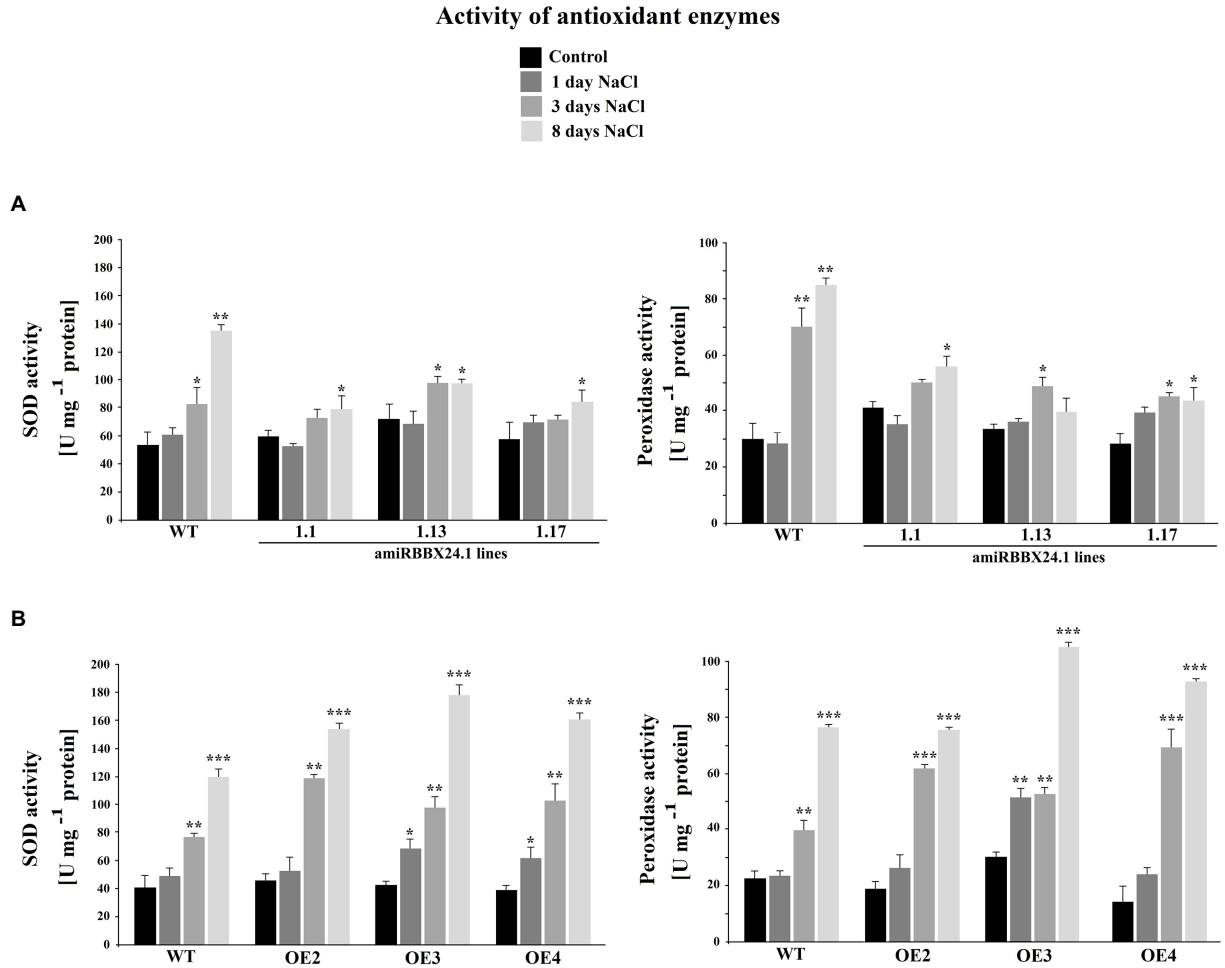
The activity of superoxide dismutase (SOD, EC 1.15.1.1) and peroxidase (POX, EC 1.11.17) enzymes in *StBBX24*-silenced **(A)** and -overexpressing **(B)** *S. tuberosum* lines subjected to salt stress. Antioxidant enzymes activity of phytotron-grown *S. tuberosum* WT, amiRBBX24.1.1, amiRBBX24.1.13 and amiRBBX24.1.17 plants, and *StBBX24*-OE2, *StBBX24*-OE3 and *StBBX24*-OE4 lines in high salt conditions (0.15 M NaCl). Measurement of superoxide dismutase and peroxidase activity was performed as described in Materials and methods. Error bars represent the bar errors. Statistical analysis was performed using the *t*-test (Statistica program, version 10). *, ** and ***, values significantly different from the control (C) value with *p* < 0.05, *p* < 0.01 and *p* < 0.001, respectively. WT, wild-type.

When plants were watered with a nutrient solution containing 0.15 M NaCl, there was no difference between wild-type and *StBBX24* overexpressing lines for 8 days (Figure 5C). Similarly, there were no noticeable changes between the wild type and *StBBX24-*silenced plants during the first 3 days of exposure to salt stress (Figure 5A). However, after 8 days of exposure to high salinity, pale green areas and severe chlorosis appeared in both well-expanded and old leaves of silenced plants (Figure 5A). When measuring the biomass of aerial parts and the leaf chlorophyll content following 8 days in high salt conditions, we noticed that amiRBBX24.1.17, amiRBBX24.1.13, and amiRBBX24.1.1 lines displayed reduced chlorophyll contents by *ca.* 38%, 21%, and 19%, respectively, compared to wild type and overexpressing lines (Figures 5B,D). As shown in Figure 5B, the degradation of photosynthetic structures under high salinity was associated with a reduction in the biomass of aerial parts that was significant for amiRBBX24.1.17 plants (*ca.* 25%) compared to wild type. This is illustrated by the data in Figure 5B showing that 3-week-old WT and silenced plants display similar weights of aerial parts and close chlorophyll contents, except for one line that exhibits a significantly lower content. Then, we investigated the impact of *StBBX24* silencing and overexpression on the activities of antioxidant enzymes during 8 days of exposure to salt stress. As shown in Figure 6, superoxide dismutase and peroxidase activity were stimulated under salinity in both types of transgenic lines compared to the control condition. However, the increment of their activity in plants overexpressing *StBBX24* was greater compared to amiRBBX24.1.1, amiRBBX24.1.13, and amiRBBX24.1.17 lines following 8 days in high salt conditions.

### Expression of genes encoding major Na^+^/H^+^ antiporters involved in salt tolerance

**Figure 7:**
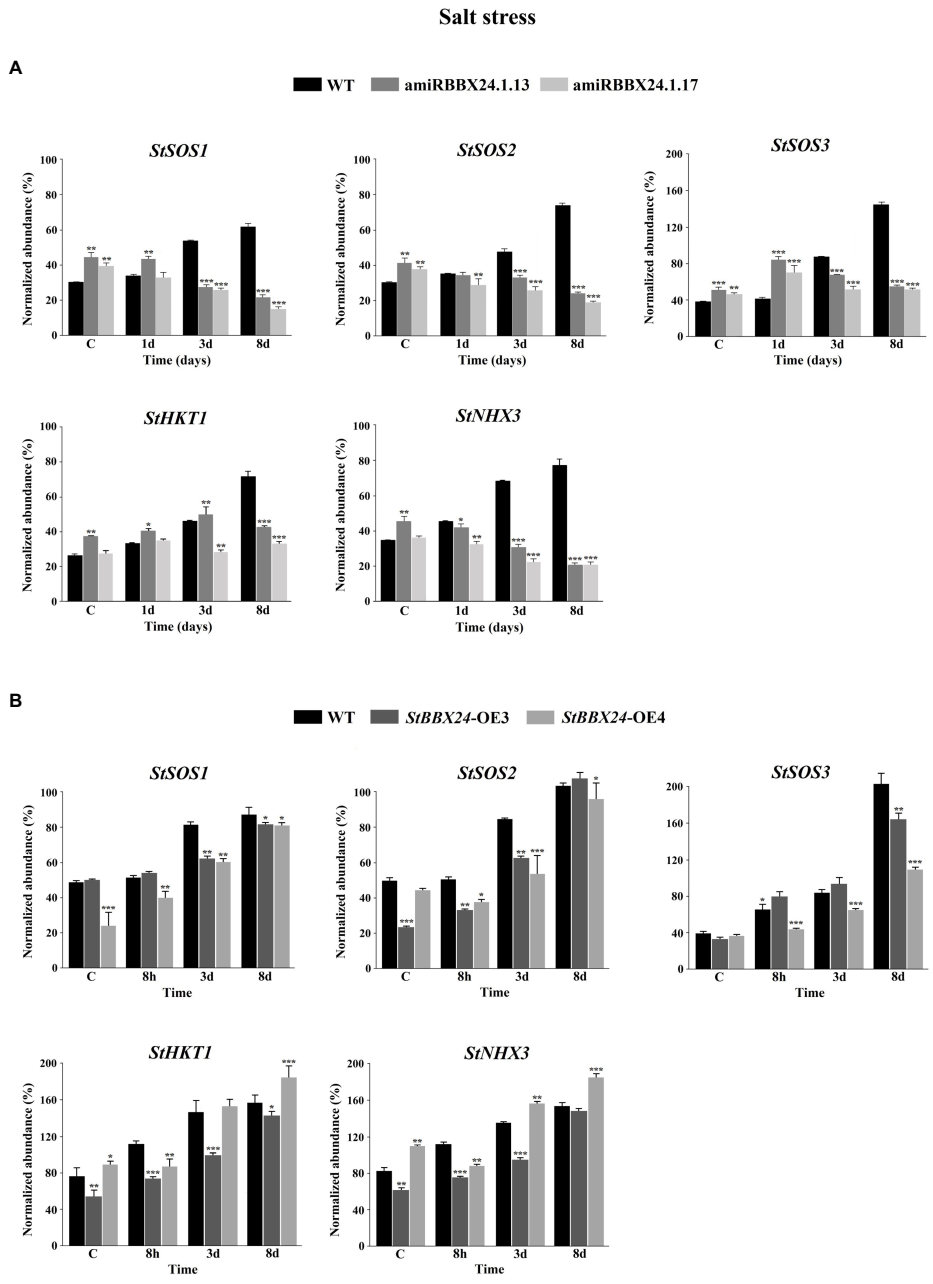
Changes in the expression of *StSOS1*, *StSOS2*, *StSOS3*, *StHKT1* and *StNHX3* genes in control conditions and plants subjected to high salt for 8 h, 3 and 8 days. **(A)** Quantitative RT-PCR analysis of *StSOS1* (ID: PGSC0003DMG400010630), *StSOS2* (ID: PGSC0003DMG400006384), *StSOS3* (ID: PGSC0003DMG400019601), *StHKT1* (ID: PGSC0003DMT400077052) and *StNHX3* (ID: PGSC0003DMG400034953) transcript abundance in leaves of 3-week-old phytotron-grown WT, amiRBBX24.1.13 and amiRBBX24.1.17 plants. **(B)** Quantitative RT-PCR analysis of *StSOS1* (ID: PGSC0003DMG400010630), *StSOS2* (ID: PGSC0003DMG400006384), *StSOS3* (ID: PGSC0003DMG400019601), *StHKT1* (ID: PGSC0003DMT400077052) and *StNHX3* (ID: PGSC0003DMG400034953) transcript abundance in leaves of 3-week-old phytotron-grown WT, *StBBX24*-OE3 and *StBBX24*-OE4 lines. Quantitative RT-PCR was performed as described in Materials and methods. Means ± SD of four values from two independent experiments (two replicates per experiment) are presented. *, ** and ***, values significantly different from the WT value (*t*-test) with *p* < 0.05, *p* < 0.01 and *p* < 0.001, respectively. WT, wild-type.

In order to gain better insight regarding the observed phenotype, we investigated the impact of *StBBX24* silencing and overexpression on the alterations in expression profile for the essential genes involved in responses to salinity stress. In our research, we focused on genes encoding Na^+^ transporters that mediate tolerance to salt stress, i.e., *HKT1* that selectively and directly unloads sodium from xylem vessels to xylem parenchyma cells, and two major factors maintaining low cytoplasmic Na^+^ concentrations in plant cells, the tonoplast-localized Na^+^/H^+^ *NHX3* exchanger and the plasma membrane Na^+^/H^+^ SALT OVERLY SENSITIVE antiporters, *SOS1*, *SOS2*, and *SOS3* (Blumwald, 2000; Deinlein et al., 2014). As shown in Figures 7A,B, substantial increases in transcript amounts of *StHKT1*, *StNHX3*, *StSOS1*, *StSOS2*, and *StSOS3* were observed in wild-type compared to control conditions, and maximal levels were reached following 8 days of salt treatment. In two silenced lines analyzed (amiRBBX24.1.13 and amiRBBX24.1.17), the transcript levels of *StHKT1*, *StNHX3*, *StSOS1*, *StSOS2*, and *StSOS3* genes were higher than in WT in control conditions. But, a gradual reduction in the abundance of *StSOS1*, *StSOS2*, and *StNHX3* transcripts was noticed from the beginning of the salt treatment, with minimal expression levels observed on the eighth day. For instance, at this stage, the *StSOS1*, *StSOS2*, and *StNHX3* transcript amounts were 3- to 4-fold lower in silenced lines than in wild-type (Figure 7A). The increased expression of *StSOS3* and *StHKT1* genes was noticed in transgenic lines compared to WT after 1 day of treatment, but then the abundance of these transcripts markedly decreased, reaching levels much lower than those in WT after 8 days of exposure (Figure 7). Significant changes in transcript accumulation were also observed in plants with *StBBX24*-OE3 and *StBBX24*-OE4 lines compared to control conditions, and maximal transcript abundance was reached on the eighth day after salt treatment (Figure 7B). These results indicate that the decreased sensitivity of silenced lines to salinity is accompanied by a much lower expression of genes encoding major Na^+^/H^+^ antiporters involved in salt tolerance.

## Discussion

In this work, we show that the clock-controlled StBBX24 protein belonging to B-box proteins in potato is associated with regulating the timing of floral induction. *Solanum tuberosum* lines silenced for *StBBX24* expression displayed much earlier flowering, while overexpression of *StBBX24* resulted in a lack of flowering or delayed production of flower buds as compared with the wild type. Furthermore, RT-qPCR analyses of *StBBX24*-silenced lines revealed that StBBX24 participates in the reproductive development of potato very likely through the regulation of the expression level of some genes essential in this process. We also reveal that silenced lines display decreased tolerance to high salinity and strongly decreased expression of genes encoding Na^+^ transporters and the activity of antioxidant enzymes. Altogether, these data unveil the role of StBBX24 in the regulation of floral induction in potato and salinity tolerance.

### StBBX24 protein regulates the timing of floral development

The phenotypic analysis of *S. tuberosum BBX24*-silenced lines showed that they exhibited early flowering as compared with WT (Figures 2A,B), while plants overexpressing *StBBX24* mostly did not produce flower buds (Figure 2C). Moreover, the transgenic plants did not display visible changes with respect to growth rate and leaves, with the exception of slight changes in tuber numbers (Figures 2A,C, Supplementary Figures S3A–C, S5A,B; Suplementary Tables S2, S3). Very interestingly, on the one hand, in Chrysanthemum, transgenic lines suppressed for *BBX24* expression also flowered earlier than WT in long-day conditions (Yang et al., 2014), while in Arabidopsis, the loss of *BBX24/STO* delayed flowering in short-day, but not, long-day conditions (Li et al., 2014). On the other hand, the same authors noted that as compared with WT plants, *BBX24*-OX Chrysanthemum lines did not exhibit delayed flowering, whereas a delay was seen when Cm-*BBX24* was overexpressed in *A. thaliana* (Li et al., 2014; Yang et al., 2014). These data reveal that BBX24 proteins play a crucial role in controlling flowering initiation in plants through a mechanism related to day length depending on the species. RT-qPCR analyses revealed that the expression of key genes in the flowering regulatory pathway was affected in *BBX24*-silenced plants (Figures 3A,B). Indeed, in these lines, the expression of genes promoting flowering, i.e., *StFKF1*, *StGI*, *StSOC1*, *StGID1*, *StSP3D*, and *StSP6A* was upregulated as compared with wild-type, while several genes encoding flowering repressors, i.e., *StSVP*, *StCDF1*, *StCDF2*, and *StSP* exhibited reduced transcript levels in full agreement with the flowering phenotype observed (Figures 3A,B). In Arabidopsis, GI and FKF1 function as circadian clock components involved in the LD-dependent degradation of CDFs that repress *CO* expression (Johansson and Staiger, 2015). The lack of StBBX24 in silenced lines, which flower earlier, correlates with the decreased expression of *StCDF* genes, indicating that a CDF-dependent mechanism regulating the expression of *CO* homolog(s) is likely to occur in potato similar to Arabidopsis. On the other hand, in Arabidopsis the expression of *FT* and *SOC1* genes is repressed by two types of factors, FLC and SVP. In potato *BBX24*-silenced plants, we observed a significant reduction of *StSVP* expression. This is associated with upregulation of floral transition genes like *StSP3D* and *StSOC1* (Figures 3A,B) that could induce *FLORAL MERISTEM IDENTITY* (*FMI*) genes. Conversely, the fact that *BBX24* overexpressing lines do not substantially modify transcript levels of some main activators and repressors of flowering, i.e., *StGI*/*StGID1* and *StCDF1*/*StCDF2* respectively, cannot support this model (Figures 4A,B). Studies aiming at isolating StBBX24-interacting partners will allow better understanding the function of BBX24 in the complex network underlying reproductive development, and provide important insights into the mode of action of B-box proteins.

In *Solanaceae*, the SELF PRUNING (SP) protein is a main repressor of flowering functioning at the level of the *SINGLE-FLOWER TRUSS* (*SFT*) gene, a homolog of the *FT* gene. In addition, note also that *SP* belongs to a separate clade of *FLOWERING TIME* regulators in *Solanaceae*, termed *CEN*/*TFL*-like (Ballerini and Kramer, 2011) and acts antagonistically to the SFT floral inducer. In the *S. tuberosum* cultivar Desirée, *StSP* expression was substantially down-regulated in *BBX24*-silenced lines. In contrast, in lines with *StBBX24* overexpression, the cyclicity of *StSP* expression was significantly affected, suggesting that, in these lines, the expression of the potato *SFT* homolog, encoding a floral inducer, could be less inhibited by SP, leading to earlier flowering. Ballerini and Kramer (2011) and Abelenda et al. (2014) suggested that *StSP3D* could be another inducer of *SFT*. Altogether, these data indicate that the early flowering phenotype of *StBBX24*-silenced lines can be associated with substantially modified expression of several genes controlling flowering. Although in this study we have focused on flowering, it is worth while mentioning that the slight changes in the tuber numbers in transformed plants as compared with the wild type, correspond to changes in transcript levels of two proteins engaged in tuber formation: StSP6A as an activator and StSP5G as a repressor of this process. Therefore, in the future, it is worth considering the potential role of StBBX24 in tuber forming as one of the factors in this complex process.

In other respects, we observed that the StBBX24 abundance was strongly decreased in leaves and shoot apex of plants exhibiting flower buds (Figures 1A,B). These data conclude that StBBX24 acts during vegetative growth as a negative regulator of genes promoting floral induction and as a positive regulator of those repressing this process, thus contributing to maintain indeterminacy of the shoot apical meristem. This role extends our knowledge about the functions of B-box proteins in plants; only a few having been described at the present time to fulfill critical roles in the control flowering. Indeed, in Arabidopsis, the key coordinator of light input is BBX1/CONSTANS/CO which promotes flowering under long-day conditions *via* triggering *FT* expression (Valverde, 2011). In potato, the BBX1 protein has been reported to fulfil a key role in tuber formation, but not in the flowering process (Abelenda et al., 2016). Our data reveal that another BBX protein in this species plays a critical function in the development of reproductive organs. Thus, we can assume that StBBX24 very likely interacts with one or several nuclear factors and modulates the expression of many genes notably involved in floral induction.

Our previous data showed that StBBX24 was localized in the cytosol and nucleus during the light phase (Kiełbowicz-Matuk et al., 2014), which was consistent with the nuclear localization reported for the Arabidopsis BBX24/STO-eGFP and Chrysanthemum Cm-BBX24-GFP fusion proteins (Indorf et al., 2007; Yang et al., 2014). Furthermore, the nuclear localization of the Arabidopsis BBX24 protein has been associated with the presence of a four-residue motif (KKPR) that acts as a functional NLS (Nuclear Localization Signal; Yan et al., 2011). Such a sequence is also present in the StBBX24 protein. Moreover, transient expression studies using Arabidopsis protoplasts have revealed nuclear localization of seven tomato BBX proteins, including SlBBX24 (Chu et al., 2016). Similarly, transgenic Arabidopsis lines expressing BBX24 proteins tagged with GFP under the control of a 35S promoter have also shown a precise nuclear localization of fusion protein (Job et al., 2018). In our work, we subsequently reveal that StBBX24 is mainly present in the chromatin fraction (Figure 1C) in accordance with its essential function in controlling gene expression at the transcript level. Because there is no DNA binding domain in StBBX24, we presume that it interacts with other proteins within the chromatin complex *via* the B-box domains and VPDLG (VP) motif, as suggested for Arabidopsis BBX24 (Gangappa and Botto, 2014). This interaction could modulate the transcriptional activity of BBX24 partners and confer BBX24 a regulatory function in transcription. In agreement with this hypothesis, several Arabidopsis BBX proteins interact with the HY5 transcription factor (ELONGATED HYPOCOTYL 5) individually or together (Datta et al., 2007, 2008; Gangappa et al., 2013). Moreover, some of them including AtBBX22, AtBBX24, and AtBBX25 interact also with HYH (HOMOLOG OF HY5) transcription factors (Datta et al., 2007, 2008; Gangappa et al., 2013).

### Role of *StBBX24* in salinity tolerance

We previously reported that *BBX24* expression is induced in Solanum plants in response to high salinity and that this induction is gated by the time of day, uncovering the interaction of photoperiod and osmotic stress in the control of *BBX24* expression (Kiełbowicz-Matuk et al., 2014). Here, we observed that *S. tuberosum* lines silenced for *StBBX24* expression exhibited severe symptoms of growth impairment under high salinity conditions (Figures 5A,B). Interestingly, the BBX24/STO (SALT TOLERANCE) protein was originally described as a protein that confers salt tolerance when it was ectopically expressed in yeast (Lippuner et al., 1996). Meanwhile, in Arabidopsis, high salinity does not induce *STO/BBX24* expression (Lippuner et al., 1996; Nagaoka and Takano, 2003). Nevertheless, overexpression of this gene enhances salt tolerance since it was found to cause increased root growth under high salinity conditions (Nagaoka and Takano, 2003). These results are consistent with our findings in silenced lines, but no evidence of salt susceptibility was observed in *Chrysanthemum morifolium* transgenic lines down-regulated for *CmBBX24* expression (Yang et al., 2014).

In this case, the very harsh treatment (400 mM NaCl for 4 weeks) compared with that here (0.15 M for 8 days) may have masked the modified response to lower salt levels. In summary, these data indicate that BBX24 proteins play an important role in response to high salinity in different plant species. However, the mechanism by which they act remains to be elucidated. Several classes of Na^+^ transporters playing major roles in the alleviation of ionic stress by sequestration of Na^+^ in vacuole (NHX), Na^+^ extrusion from cells (SOS1, 2 and 3) and facilitated Na^+^ circulation (Class 1 HKT1) have been characterized (Blumwald, 2000; Sunarpi et al., 2005; Yamaguchi et al., 2013; Deinlein et al., 2014). Our results revealed that *S. tuberosum* lines silenced for *StBBX24* displayed strongly decreased expression of all these genes following 8 days of salt application concomitantly to the stress symptoms appearing on leaves (Figure 5A). On the contrary, a substantial increase in the transcript abundance of these transporters was noticed in WT plants in response to salt consistent with their preserved growth (Figure 7). These findings are in agreement with reports showing improved salt tolerance in transgenic Arabidopsis, tomato, apple, poplar, Chrysanthemum and barley lines overexpressing *SOS2*, *SOS1*, or *NHX* (Guo et al., 2004; Huertas et al., 2012; Wang et al., 2012; Adem et al., 2014; An et al., 2014; Yang et al., 2015). Thus, our data revealing that the expression of these transporters is compromised in potato in the absence of StBBX24 indicate that the mechanisms leading to ion homeostasis under salinity are impaired, consequently causing inhibition of many cellular and photosynthetic processes due to Na^+^ over-accumulation within all cell sub-compartments. They prompt us to propose that StBBX24 is a down-stream component in the signaling pathway triggered by high salinity and regulating the expression of the major genes involved in cell sodium homeostasis.

## Conclusion

Our results characterized the StBBX24 protein as an essential component in the network controlling reproductive development and in salt responses. One major aim for future research will be to identify the signals and actors involved in *StBBX24* expression. We recently reported that the circadian rhythmicity of its expression was maintained by the StZPR1 nuclear factor that plays a fine tuning role downstream in the clock signaling network (Kiełbowicz-Matuk et al., 2017). The presence of various potential *cis*-elements associated with light, circadian, hormones or stress responses in the promoter region of *StBBX24* suggests that multiple factors participate in the regulation of its expression. In other respects, studies aiming at isolating StBBX24-interacting partners will allow better understanding the function of BBX24 in the complex network underlying reproductive development and stress responses, and provide important insights into the mode of action of B-box proteins.

## Data availability statement

The raw data supporting the conclusions of this article will be made available by the authors, without undue reservation.

## Author contributions

AK-M and TR: conceptualization and funding acquisition. KG, MB, UT, JC, and AK-M: performed the experiments. AK-M, UT, and TR: writing–original draft preparation. AK-M, KG, and UT: writing–review and editing. AK-M, KG, UT, and MB: visualization. All authors contributed to the article and approved the submitted version.

## Funding

This work was supported by the Polish National Science Centre under grants no. 2014/15/B/NZ9/04809 and 2018/29/B/NZ9/01457.

## Conflict of interest

The authors declare that the research was conducted in the absence of any commercial or financial relationships that could be construed as a potential conflict of interest.

## Publisher’s note

All claims expressed in this article are solely those of the authors and do not necessarily represent those of their affiliated organizations, or those of the publisher, the editors and the reviewers. Any product that may be evaluated in this article, or claim that may be made by its manufacturer, is not guaranteed or endorsed by the publisher.

## Footnotes

^1^ www.wmd3.weigelworld.org
