# Corrigendum: The StBBX24 protein affects the floral induction and mediates salt tolerance in *Solanum tuberosum*

**DOI:** 10.3389/fpls.2024.1422048

**Published:** 2024-05-08

**Authors:** Agnieszka Kiełbowicz-Matuk, Klaudia Grądzka, Magdalena Biegańska, Urszula Talar, Jagoda Czarnecka, Tadeusz Rorat

**Affiliations:** Department of Regulation of Gene Expression, Institute of Plant Genetics, Polish Academy of Sciences, Poznan, Poland

**Keywords:** antioxidant enzymes, double B-box protein, flowering time, flowering-related genes, salinity, sodium transporters, *Solanum tuberosum*

Incorrect funding

In the published article, there was an error in the Funding statement. The number of the second project from which some of the studies included in the publication were carried out was omitted. The correct Funding statement appears below.

Funding

This work was supported by the Polish National Science Centre under grants no. 2014/15/B/NZ9/04809 and 2018/29/B/NZ9/01457.

The authors apologize for this error and state that this does not change the scientific conclusions of the article in any way. The original article has been updated.

