# Supplementary material for: The StBBX24 protein affects the floral induction and mediates salt tolerance in Solanum tuberosum
Source: Front Plant Sci. 2022 Sep 7;13:965098. doi: 10.3389/fpls.2022.965098 (PMC9490078; doi:10.3389/fpls.2022.965098)
Supplement: Supplementary file 1 [file Data_Sheet_1.PDF]

Supplementary Tables

Table S1. The oligonucleotide primers used.

**Nucleotide sequence of amiRs and primers used during expression construct preparation generating artificial microRNA**	
**amiRNABBX24.1**	5′ -CAGCATTTGACTGTCCACATT- 3′
**amiRNABBX24.2**	5′ -GTGGATCATTGTCAACGATAG- 3′
**amiRNABBX24.3**	5′ -AGGACAAAGTGATGAGGTATC- 3′
**AMIR319bXhoI-Fd**	5′ -AACTCGAGATCTCTTCTTCTCTCATCTCTCTTT- 3′
**BMIR319bHindIII-Rev**	5′ -CCAAGCTTTTACAAATAAGTCATCTTTTATTGAG- 3′
**I-amiRNABBX24.1**	5′ -GCCTGTGATTTGCTGTGCTGATAATTTTTATCTTTCTCA- 3′
**II-amiRNABBX24.1**	5′ -ATTATCAGCACAGCAAATCACAGGCATAGAGAAGTGAAGA- 3′
**III-amiRNABBX24.1**	5′ -GATACCAGCACAGCATATCACAGTCATGGAGTAATATGTG- 3′
**IV-amiRNABBX24.1**	5′ -GACTGTGATATGCTGTGCTGGTATCCTCCACCAACTCAAC- 3′
**I-amiRNABBX24.2**	5′ -GCAAGCAGTAAAAAACCAACTGAATTTTTATCTTTCTCA-3′
**II-amiRNABBX24.2**	5′ -ATTCAGTTGGTTTTTTACTGCTTGCATAGAGAAGTGAAGA-3′
**III-amiRNABBX24.2**	5′ -GATCCGTTGGTTTTTAACTGCTTTCATGGAGTAATATGTG- 3′
**IV-amiRNABBX24.2**	5′ -GAAAGCAGTTAAAAACCAACGGATCCTCCACCAACTCAAC- 3′
**I-amiRNABBX24.3**	5′ -GCAAGAAGCCCAGATTTGAAATAATTTTTATCTTTCTCA- 3′
**II-amiRNABBX24.3**	5′ -ATTATTTCAAATCTGGGCTTCTTGCATAGAGAAGTGAAGA- 3′
**III-amiRNABBX24.3**	5′ -GATACTTCAAATCTGCGCTTCTTTCATGGAGTAATATGTG- 3′
**IV-amiRNABBX24.3**	5′ -GAAAGAAGCGCAGATTTGAAGTATCCTCCACCAACTCAAC- 3′
**Primers used for cDNA fragments amplification of the analyzed genes from *S. tuberosum* during real-time PCR reactions**	
**StBBX24qRT-Fd**	5′ -CAAGCTGCAATAAGGAAG- 3′
**StBBX24qRT-Rev**	5′ -CTCGACTCATAATCTGGAA- 3′
**StFKF1qRT-Fd**	5′ -AAAGAAACTTGCCAGCCTCA- 3′
**StFKF1qRT-Rev**	5′ -GATGCAACATCCCTTGGAGT- 3′
**StGIqRT-Fd**	5′ -GGTCCCGGGTTTCTTAGACT- 3′
**StGIqRT-Rev**	5′ -CATGGTTGCAATTCCCTCTC- 3′
**StSOC1qRT-Fd**	5′ -AATTCAACGAGCAGGCAAGT- 3′
**StSOC1qRT-Rev**	5′ -TTGTCCACCGTCATTGTTTC- 3′
**StGID1qRT-Fd**	5′ -GTCCCTTCAACCCCAGAAAT- 3′
**StGID1qRT-Rev**	5′ -AAAAATCCACCACCATGGAA- 3′
**StEBSqRT-Fd**	5′ -GGACTGATTCCTTGCCAAAA- 3′
**StEBSqRT-Rev**	5′ -AGGCGTTAAGCACCTTTGAA- 3′
**StSVPqRT-Fd**	5′ -AAGGCAAATGAGGGGAGAA- 3′
**StSVPqRT-Rev**	5′ -ATGCCCTTTTGTTGGAGTTG- 3′
**StCDF1qRT-Fd**	5′ -GCATCTAGGAGCCGTGAGAC- 3′
**StCDF1qRT-Rev**	5′ -GAATCTGACCACGCGAATTT- 3′
**StCDF2qRT-Fd**	5′ -TCATCGTTCACGCATCTCTC- 3′
**StCDF2qRT-Rev**	5′ -AGATCGGTTCACGGAGCTAA- 3′
**StSP6AFd**	5′ -GTTCATATTGGAGGGGACGA- 3′
**StSP6ARev**	5′ -TCCCATCGTAGGTGTTGGAT- 3′
**StSP3DFd**	5′ -TGGTTATGGTGGACCCAGAT- 3′
**StSP3DRev**	5′ -GCCATCCTGGAGCATACACT- 3′
**StSP5GFd**	5′ -CCAAACCCTAGCAATCCAAA- 3′
**StSP5GRev**	5′ -TTGCTGGAACAACACGAAAA- 3′
**StSPFd**	5′ -GGGTTGAAGTTCATGGTGGT- 3′
**StSPRev**	5′ -CAATATTTGGCCTTGGCATT- 3′
**StSOS1Fd**	5′ -CATGGTCAATTATGCAAAGCA- 3′
**StSOS1Rev**	5′ -AGTCTGGATGCAGTTGCTGA- 3′
**StSOS2Fd**	5′ -TGAAAGTGTCGCCATGAAAG- 3′
**StSOS2Rev**	5′ -AGATCTTCGTGCGAGTTGCT- 3′
**StSOS3Fd**	5′ -TTCCACCCTAATGCACCAGT- 3′
**StSOS3Rev**	5′ -TGTCTGCATCGCTAAACGTC - 3′
**StNHX3Fd**	5′ -CGTTCGTGAAATATCGCTCA- 3′
**StNHX3Rev**	5′ -ATCCATCCCCACGTACAAAA- 3′
**StHKT1Fd**	5′ -GCGCGAAACAAATCCTTAAC- 3′
**StHKT1Rev**	5′ -AGGCGTAAACAAGGAGCAAA- 3′

All restriction sites used in vector construction are underlined.

Table S2. Tubers yield (shape index, weight and the number) in WT and *S. tuberosum* lines silenced and overexpressed *StBBX24*.

Phenotype	Tubers shape index	Tuber weight [g]	The number of tubers
WT	1.37 (±0.17)	19.52 (±14.45)	59
StBBX24-OE3	1.46*(±0.19)	18.27 (±10.52)	61
StBBX24-OE4	1.39 (±0.2)	19.02 (±12.38)	60
amiRBBX24.1.1	1.4 (±0.17)	15.60*(±10.59)	69**
amiRBBX24.1.5	1.51*(±0.18)	16.12*(±10.81)	71**
amiRBBX24.1.9	1.52*(±0.18)	17.10*(±12.2)	73**
amiRBBX24.1.13	1.62*(±0.2)	17.67*(±12.05)	69**
amiRBBX24.1.17	1.55*(±0.22)	17.41*(±10.9)	70**

± Standard deviation (SD). Data represent the average of five pots (twenty plants) for each line.*, ** and ***, values significantly different from the WT value (*t*-test) with $p < 0.05$, $p < 0.01$ and $p < 0.001$, respectively.

Wild-type plants (WT) and amiRBBX24.1.1, amiRBBX24.1.5, amiRBBX24.1.9, amiRBBX24.1.13, amiRBBX24.1.17, *StBBX24*-OE3 and *StBBX24*-OE4 transgenic lines grown in the growth room in standard conditions (20°C±1, 40 % relative humidity and PFD of 350 µmoles photons m^{-2} s^{-1}) under a 16-h photoperiod for three months. Tubers from twenty plants per genotype (four plants per plastic pot) were analyzed.

Table S3. Morphological characteristics of *S. tuberosum* lines silenced and overexpressed *StBBX24*.

Phenotype	Stem length	Internode length	Number of nodes	Number of leaves
WT	12.6 (±0.12)	3.8 (±0.11)	3-4	8-10
StBBX24-OE3	11.6 (±0.19)	3.4 (±0.12)	2-3	6-8
StBBX24-OE4	12.5 (±0.12)	3.6 (±0.13)	3-4	8-10
amiRBBX24.1.1	11.2 (±0.13)	3.2 (±0.15)	3-4	6-8
amiRBBX24.1.5	13.1 (±0.14)	3.8 (±0.13)	3-4	8-10
amiRBBX24.1.9	14.5 (±0.12)	3.9 (±0.15)	3-4	9-10
amiRBBX24.1.13	14.2 (±0.18)	4.2 (±0.16)	2-3	9-10
amiRBBX24.1.17	13.4 (±0.12)	3.7 (±0.17)	3-4	8-10

± Standard deviation (SD). Data represent the average of five pots (twenty plants) for each line.*, ** and ***, values significantly different from the WT value (*t*-test) with $p < 0.05$, $p < 0.01$ and $p < 0.001$, respectively.

Wild-type plants (WT) and amiRBBX24.1.1, amiRBBX24.1.5, amiRBBX24.1.9, amiRBBX24.1.13, amiRBBX24.1.17, *StBBX24*-OE3 and *StBBX24*-OE4 transgenic lines grown in the growth room in standard conditions (20°C±1, 40 % relative humidity and PFD of 350 µmoles photons m^{-2} s^{-1}) under a 14-h photoperiod for three weeks. Twenty plants per genotype (four plants per plastic pot) were analyzed.
