# Supplementary material for: The StBBX24 protein affects the floral induction and mediates salt tolerance in Solanum tuberosum
Source: Front Plant Sci. 2022 Sep 7;13:965098. doi: 10.3389/fpls.2022.965098 (PMC9490078; doi:10.3389/fpls.2022.965098)
Supplement: Supplementary file 2 [file Data_Sheet_2.PDF]

Supplementary Figures

Figure S1.

(A)

```
StBBX24.1  ATGAAGATCCAGTGTGATGTGTGTGAGAAAGCTCAAGCTACTGTGATTTGCTGTGCTGATGAGGCTGCTTTGTGTGCAAAATGTGATATTGAAGTTCATG   100
StBBX24.2  ATGAAGATCCAGTGTGATGTGTGTGAGAAAGCTCAAGCTACTGTGATTTGCTGTGCTGATGAGGCTGCTTTGTGTGCAAAATGTGATATTGAAGTTCATG   100
StBBX24.3  ATGAAGATCCAGTGTGATGTGTGTGAGAAAGCTCAAGCTACTGTGATTTGCTGTGCTGATGAGGCTGCTTTGTGTGCAAAATGTGATATTGAAGTTCATG   100
StBBX24.4  ATGAAGATCCAGTGTGATGTGTGTGAGAAAGCTCAAGCTACTGTGATTTGCTGTGCTGATGAGGCTGCTTTGTGTGCAAAATGTGATATTGAAGTTCATG   100

StBBX24.1  CTGCTAATAAATTAGCAAGTAAGCATCAGAGGCTTCATCTTCAGTGCCTATCTAACAAGCTTCCTCCTTGTGATATTTGCCAAGATAAAGCAGCCTTCAT   200
StBBX24.2  CTGCTAATAAATTAGCAAGTAAGCATCAAAGGCTTCATCTTCAGTGCCTATCTAACAAGCTTCCTCCTTGTGATATTTGCCAAGATAAAGCAGCCTTCAT   200
StBBX24.3  CTGCTAATAAATTAGCAAGTAAGCATCAAAGGCTTCATCTTCAGTGCCTATCTAACAAGCTTCCTCCTTGTGATATTTGCCAAGATAAAGCAGCCTTCAT   200
StBBX24.4  CTGCTAATAAATTAGCAAGTAAGCATCAGAGGCTTCATCTTCAGTGCCTATCTAACAAGCTTCCTCCTTGTGATATTTGCCAAGATAAAGCAGCCTTCAT   200

StBBX24.1  CTTCTGTGTTGAGGATAGAGCTCTCTTTTGCAAGGACTGTGACGAAGCAATTCATTCAGCCAGCAGCCTCGCCAAGAACCACCAACGCTTCTTAGCCACT   300
StBBX24.2  CTTCTGTGTTGAGGATAGAGCTCTCTTTTGCAAGGACTGTGACGAAGCAATTCATTCAGCCAGCAGCCTCGCTAAGAACCACCAACGCTTCTTAGCCACT   300
StBBX24.3  CTTCTGTGTTGAGGATAGAGCTCTCTTTTGCAAGGACTGTGACGAAGCAATTCATTCAGCCAGCAGCCTTGCCAAGAACCACCAACGCTTCTTAGCCACT   300
StBBX24.4  CTTCTGTGTTGAGGATAGAGCTCTCTTTTGCAAGGACTGTGACGAAGCAATTCATTCAGCCAGCAGCCTCGCCAAGAACCACCAACGCTTCTTAGCCACT   300

StBBX24.1  GGAATCCGTGTAGCCTTGAGCTCAAGCTGCAATAAGGAAGCAGTAAAAAACCAACTGGAGCCACAACCACCTCAGCAGAATTCTCAACAAGTTGGCTTGA   400
StBBX24.2  GGAATCCGTGTAGCCTTGAGCTCAAGCTGCAATAAGGAAGCAGTAAAAAACCAACTGGAGCCACAACCACCTCAGCAGAATTCCCAACAAGTTGGCTTGA   400
StBBX24.3  GGAATCCGTGTAGCCTTGAGCTCAAGCTGCAATAAGGAAGCAGTAAAAAACCAACTGGAGCCACAACCACCTCAGCAGAATTCTCAACAGGTTGGCTTGA   400
StBBX24.4  GGAATCCGTGTAGCCTTGAGCTCAAGCTGCAATAAGGAAGCAGTAAAAAACCAACTGGAGCCACAACCACCTCAGCAGAATTCTCAACAAGTTGGCTTGA   400

StBBX24.1  AAATGCCTACGCAGCAATTGTCTGGTATCACATCACCATCTTGGCCTGTCGATGATTTACTAGAATTTCCAGATTATGAGTCGAGTGACAAGAAGGATCT   500
StBBX24.2  AAATGCCTCCACAGCAATTGTCCGGTATCACATCACCATCTTGGCCTGTTGATGATTTACTAGGATTTCCAGATTATGAGTCGAGTGACAAGAAGGATCT   500
StBBX24.3  AAATGCCTCCACAGCAATTGTCCGGTATCACATCACCATCTTGGCCTGTCGATGATTTACTAGAATTTCCAGATTATGAGTCGAGTGACAAGAAGGATCT   500
StBBX24.3  AAATGCCTACGCAGCAATTGTCTGGTATCACATCACCATCTTGGCCTGTCGATGATTTACTAGGATTTCCAGATTATGAGTCTAGTGACAAGAAGGATCT   500

StBBX24.1  ACTTGAGCTTGGTGAATTTGAGTGGTTAGTAGGCATTGATCTCTTTGGTGAACAAACAGCAGCTGAAGTACCTGAGCTATCAGTACCTCAGTCGAGCAAC   600
StBBX24.2  ACTTGAGCTTGGTGAATTTGAGTGGCTAGGAGGCATTGATCTCTTTGGTGAACAAACAGCAGCTGAAGTACCTGAGCTATCAGTACCTCAGTCGAGCAAC   600
StBBX24.3  ACTTGAGCTTGGTGAATTTGAGTGGTTAGGAGGCATTGATCTCTTTGGTGAACAAACAGCAGCTGAAGTACCTGAGCTATCAGTACCTCAGTCGAGCAAC   600
StBBX24.4  ACTTGAGCTTGGTGAATTTGAGTGGTTAGGAGGCATTGATCTCTTTGGTGAACAAACAGCAGCTGAAGTACCCGAGCTATCAGTACCTCAGTCGAGCAAC   600

StBBX24.1  ACAAATATTTACAGGACAACCAAATATCAAATGCCTTACAAGAAGCCCAGATTTGAAATCCCAGATGAAGATGAGTATTTTACTGTCCCAGATCTTGGTTGA 700
StBBX24.2  ACAAATATTTACAGGACAACCAAATATCAAATGCCTTACAAGAAGCCCAGATTTGAAATCCCAGATGAAGATGAGTATTTTACTGTCCCAGATCTTGGTTGA 700
StBBX24.3  ACAAATATTTACAGGACAACCAAATATCAAATGCCTTACAAGAAGCCCAGATTTGAAATCCCAGATGAAGATGAGTATTTTACTGTCCCAGATCTTGGTTGA 700
StBBX24.4  ACAAATATTTACAGGACAACCAAATATCAAATGCCTTACAAGAAGCCCAGATTTGAAATCCCAGATGAAGATGAGTATTTTACTGTCCCAGATCTTGGTTGA 700
```

(B)

```
StBBX24.1  MKIQCDVCEKAQATVICCADEAALCAKCDIEVHAANKLASKHQRLHLQCLSNKLPPCDICQDKAAFIFCVEDRALFCKDCDEAIHSASSLAKNHQRFLAT  100
StBBX24.2  MKIQCDVCEKAQATVICCADEAALCAKCDIEVHAANKLASKHQRLHLQCLSNKLPPCDICQDKAAFIFCVEDRALFCKDCDEAIHSASSLAKNHQRFLAT  100
StBBX24.3  MKIQCDVCEKAQATVICCADEAALCAKCDIEVHAANKLASKHQRLHLQCLSNKLPPCDICQDKAAFIFCVEDRALFCKDCDEAIHSASSLAKNHQRFLAT  100
StBBX24.4  MKIQCDVCEKAQATVICCADEAALCAKCDIEVHAANKLASKHQRLHLQCLSNKLPPCDICQDKAAFIFCVEDRALFCKDCDEAIHSASSLAKNHQRFLAT  100

StBBX24.1  GIRVALSSSCNKEAVKNQLEPQPPQQNSQQVGLKMPTQQLSGITSPSWPVDDLLEFPDYESSDKKDLLELGEFEWLVGIDLFGEQTAAEVPELSVPQSSN  200
StBBX24.2  GIRVALSSSCNKEAVKNQLEPQPPQQNSQQVGLKMPPQQLSGITSPSWPVDDLLGFPDYESSDKKDLLELGEFEWLGGIDLFGEQTAAEVPELSVPQSSN  200
StBBX24.3  GIRVALSSSCNKEAVKNQLEPQPPQQNSQQVGLKMPPQQLSGITSPSWPVDDLLEFPDYESSDKKDLLELGEFEWLGGIDLFGEQTAAEVPELSVPQSSN  200
StBBX24.4  GIRVALSSSCNKEAVKNQLEPQPPQQNSQQVGLKMPTQQLSGITSPSWPVDDLLGFPDYESSDKKDLLELGEFEWLGGIDLFGEQTAAEVPELSVPQSSN  200

StBBX24.1  TNIYRTTKYQMPYKKPRFEIPDEDEYFTVPDLG-
StBBX24.2  TNIYRTTKYQMPYKKPRFEIPDEDEYFTVPDLG-
StBBX24.3  TNIYRTTKYQMPYKKPRFEIPDEDEYFTVPDLG-
StBBX24.4  TNIYRTTKYQMPYKKPRFEIPDEDEYFTVPDLG-
```

Figure S1. A comparison of the cDNA sequences of the *StBBX24* gene alleles **(A)** and the protein sequence **(B)** from the potato cultivar Desirée. The nucleotide and amino acid differences between the sequences are marked in red. Two putative conserved B-Box domains, B1 and B2 and the VPDLG motif, are marked in yellow. The START and STOP codons are underlined and bolded. The nucleotide sequences chosen as targets for the artificial micro RNA are shadowed.

Figure S2.

(A)

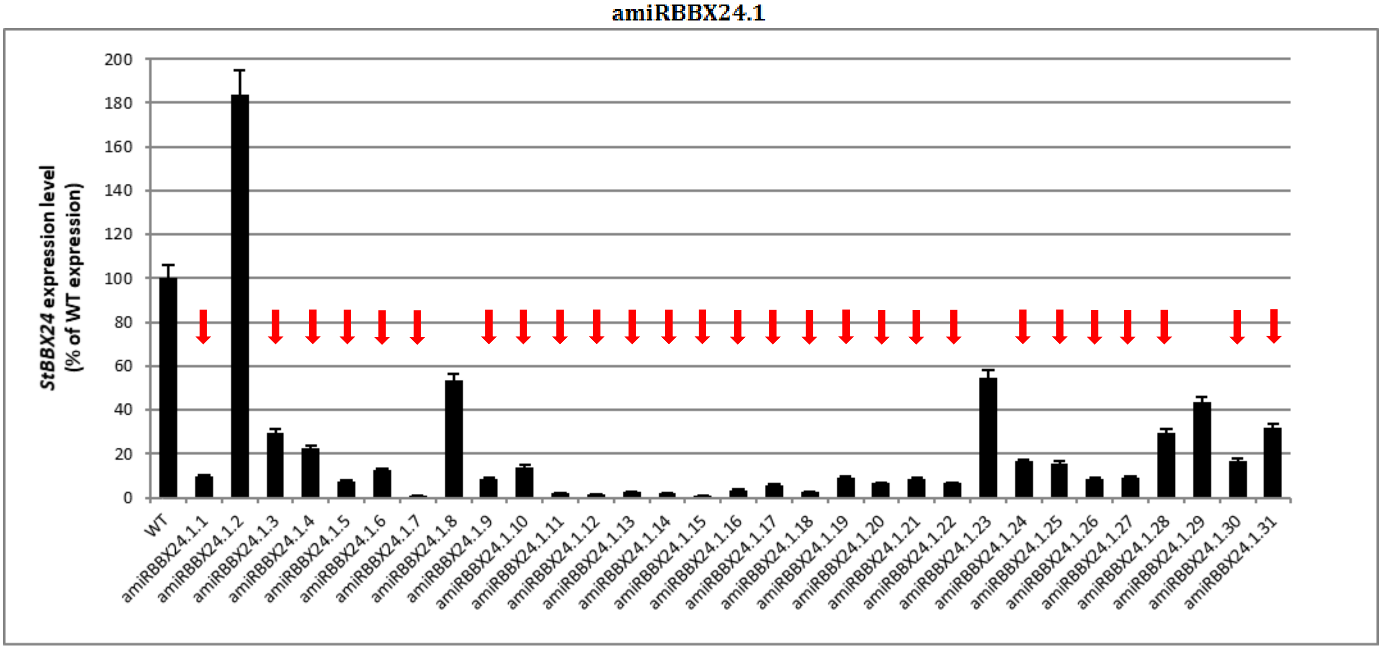
amiRBBX24.1
StBBX24 expression level (% of WT expression)
0
20
40
60
80
100
120
140
160
180
200
WT
amiRBBX24.1.1
amiRBBX24.1.2
amiRBBX24.1.3
amiRBBX24.1.4
amiRBBX24.1.5
amiRBBX24.1.6
amiRBBX24.1.7
amiRBBX24.1.8
amiRBBX24.1.9
amiRBBX24.1.10
amiRBBX24.1.11
amiRBBX24.1.12
amiRBBX24.1.13
amiRBBX24.1.14
amiRBBX24.1.15
amiRBBX24.1.16
amiRBBX24.1.17
amiRBBX24.1.18
amiRBBX24.1.19
amiRBBX24.1.20
amiRBBX24.1.21
amiRBBX24.1.22
amiRBBX24.1.23
amiRBBX24.1.24
amiRBBX24.1.25
amiRBBX24.1.26
amiRBBX24.1.27
amiRBBX24.1.28
amiRBBX24.1.29
amiRBBX24.1.30
amiRBBX24.1.31

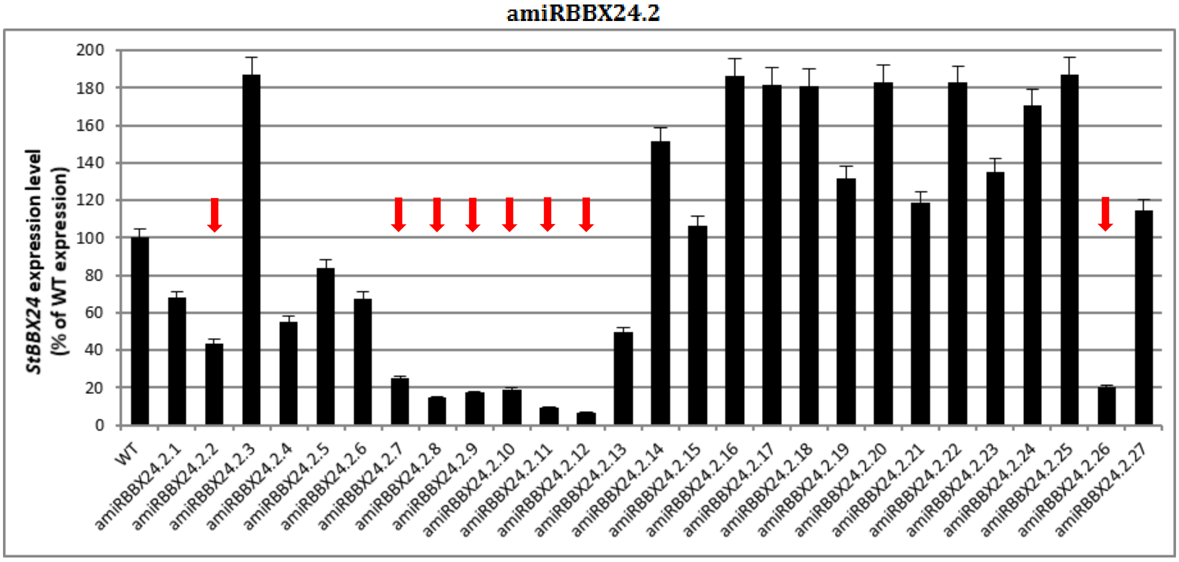
amiRBBX24.2
StBBX24 expression level (% of WT expression)
0
20
40
60
80
100
120
140
160
180
200
WT
amiRBBX24.2.1
amiRBBX24.2.2
amiRBBX24.2.3
amiRBBX24.2.4
amiRBBX24.2.5
amiRBBX24.2.6
amiRBBX24.2.7
amiRBBX24.2.8
amiRBBX24.2.9
amiRBBX24.2.10
amiRBBX24.2.11
amiRBBX24.2.12
amiRBBX24.2.13
amiRBBX24.2.14
amiRBBX24.2.15
amiRBBX24.2.16
amiRBBX24.2.17
amiRBBX24.2.18
amiRBBX24.2.19
amiRBBX24.2.20
amiRBBX24.2.21
amiRBBX24.2.22
amiRBBX24.2.23
amiRBBX24.2.24
amiRBBX24.2.25
amiRBBX24.2.26
amiRBBX24.2.27

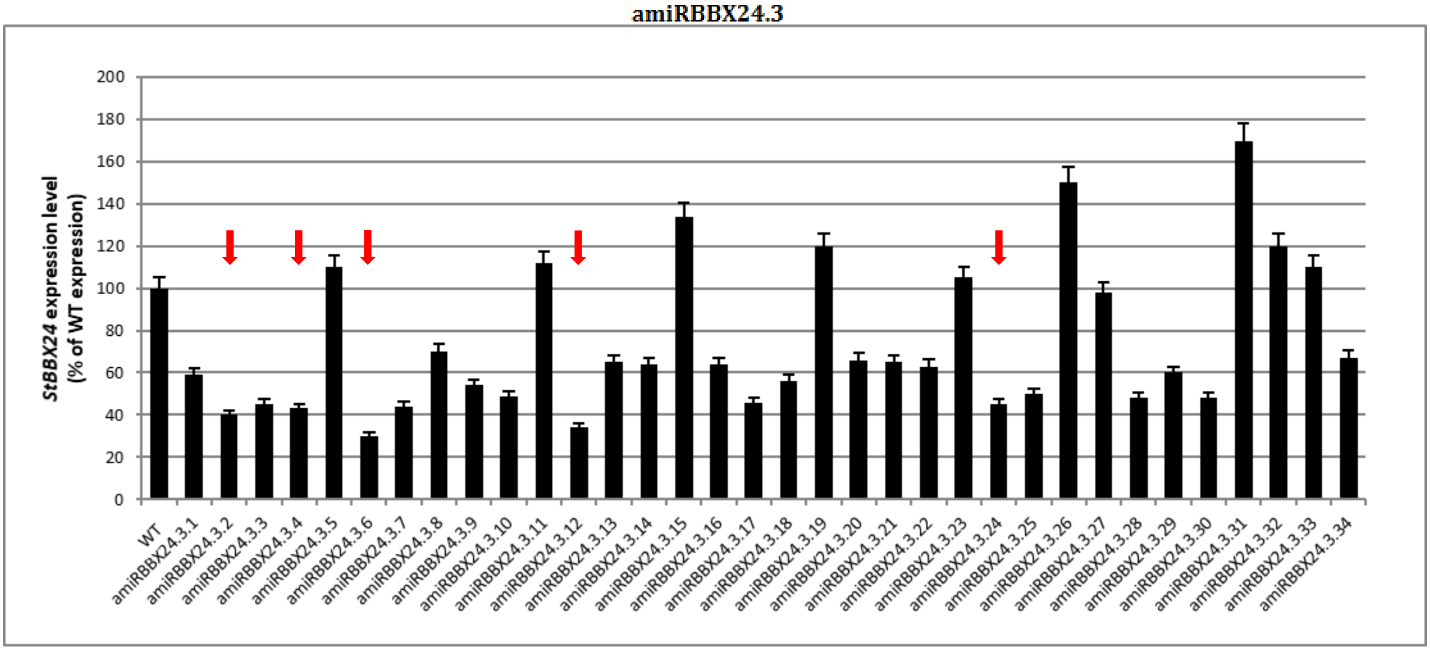
amiRBBX24.3
StBBX24 expression level (% of WT expression)
0
20
40
60
80
100
120
140
160
180
200
WT
amiRBBX24.3.1
amiRBBX24.3.2
amiRBBX24.3.3
amiRBBX24.3.4
amiRBBX24.3.5
amiRBBX24.3.6
amiRBBX24.3.7
amiRBBX24.3.8
amiRBBX24.3.9
amiRBBX24.3.10
amiRBBX24.3.11
amiRBBX24.3.12
amiRBBX24.3.13
amiRBBX24.3.14
amiRBBX24.3.15
amiRBBX24.3.16
amiRBBX24.3.17
amiRBBX24.3.18
amiRBBX24.3.19
amiRBBX24.3.20
amiRBBX24.3.21
amiRBBX24.3.22
amiRBBX24.3.23
amiRBBX24.3.24
amiRBBX24.3.25
amiRBBX24.3.26
amiRBBX24.3.27
amiRBBX24.3.28
amiRBBX24.3.29
amiRBBX24.3.30
amiRBBX24.3.31
amiRBBX24.3.32
amiRBBX24.3.33
amiRBBX24.3.34

(B)

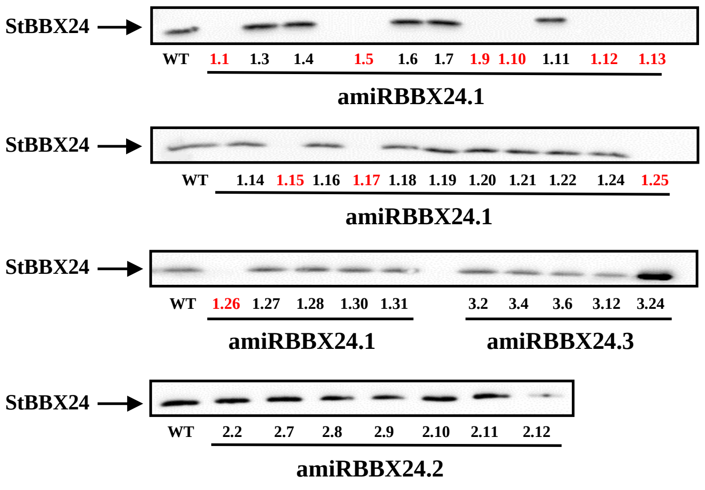

Figure S2. Artificial miRNAs silence the expression of the *StBBX24* gene in *S. tuberosum* cv. Desirée. Individual plants from transgenic lines were tested for their ability to silence *StBBX24* gene expression.

(A) Quantitative RT-PCR measurements of the *StBBX24* transcript level in selected transgenic lines (determined as a percentage of *StBBX24* transcript level in the control potato plants). The arrows indicate transgenic lines selected to Western blot analysis. WT - wild type.

(B) Western blot analysis of the presence of StBBX24 protein in the control Desirée plants and the selected transgenic lines. WT - wild type.

Figure S3.

(A)

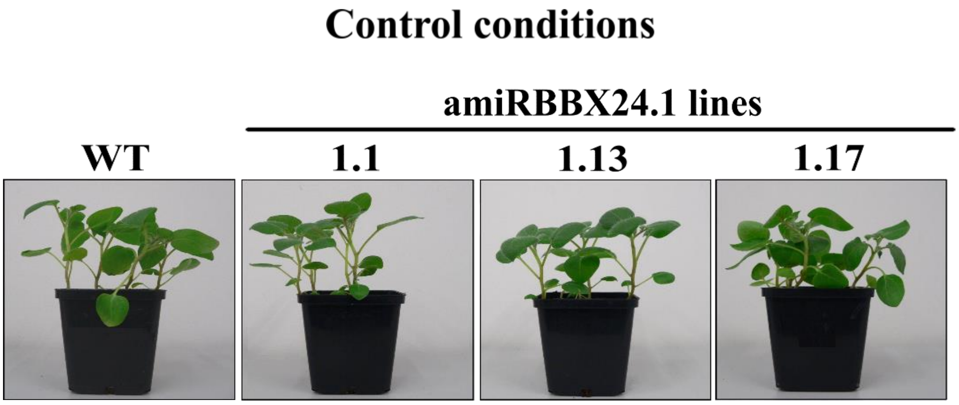

(B)

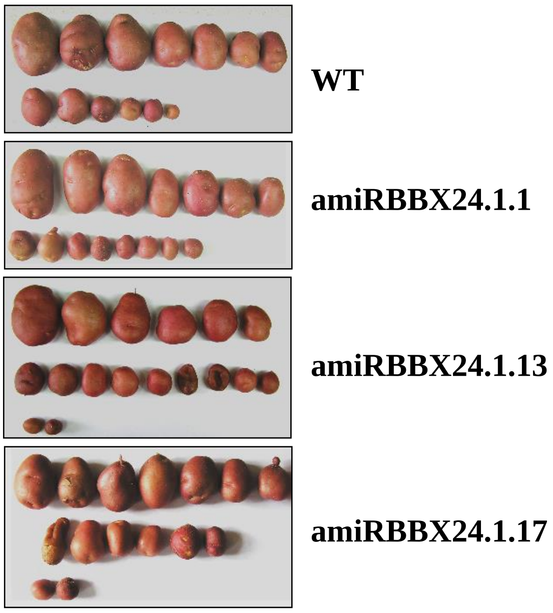

(C)

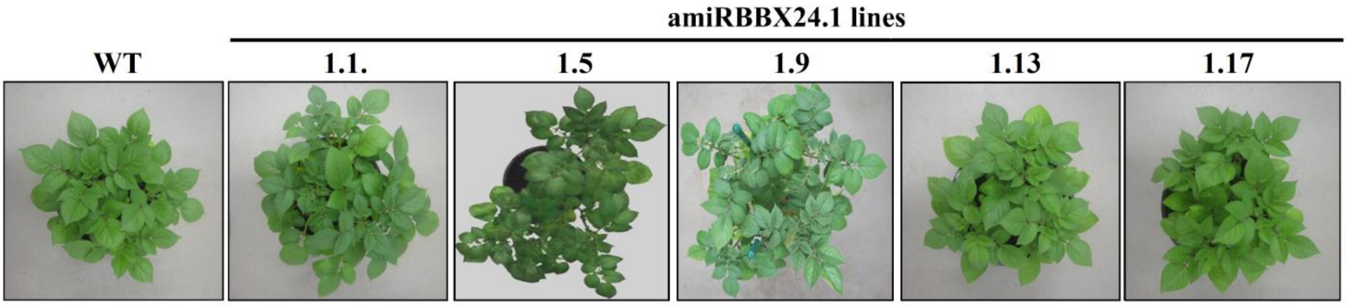

Figure S3. Phenotype of *S. tuberosum* plants silenced for *StBBX24* expression.

(A) Phenotype of WT and amiRBBX24.1.1, amiRBBX24.1.13 and amiRBBX24.1.17 transgenic plants grown in standard conditions (20°C±1, 40 % relative humidity and PFD of 350 µmoles photons m^{-2} s^{-1}) under a 14-h photoperiod for 3 weeks. WT - wild type.

(B) Tubers yield in WT, amiRBBX24.1.1, amiRBBX24.1.13 and amiRBBX24.1.17 transgenic plants grown in the growth room in standard conditions (20°C±1, 40 % relative humidity and PFD of 350 µmoles photons m^{-2} s^{-1}) under a 16-h photoperiod for 3 months. WT - wild type.

(C) Phenotype of WT, amiRBBX24.1.1, amiRBBX24.1.5, amiRBBX24.1.9, amiRBBX24.1.13 and amiRBBX24.1.17 transgenic plants grown in the growth room in standard conditions (20°C±1, 40 % relative humidity and PFD of 350 µmoles photons m^{-2} s^{-1}) under a 12-h photoperiod for 3 months. WT - wild type.

Figure S4.

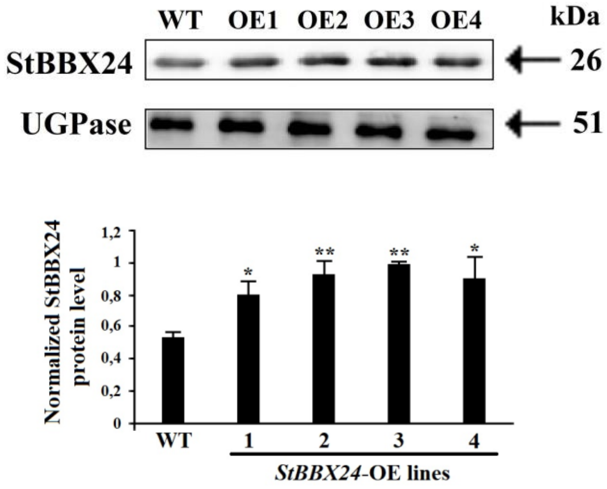

Figure S4. The StBBX24 protein abundance in four *S. tuberosum* overexpressed lines. Individual plants from transgenic lines were tested for the StBBX24 protein level using Western blot analysis. Western analysis of UDP-glucosepyrophosphorylase (UGPase) abundance was performed using cytosolic proteins and UGPase antibody to insure the equal loading of all lanes. Means ± SD of 3 values from two independent experiments (two replicates per experiment) are presented. WT - wild type.

Figure S5.

(A)

Control conditions

***StBBX24*-OE lines**

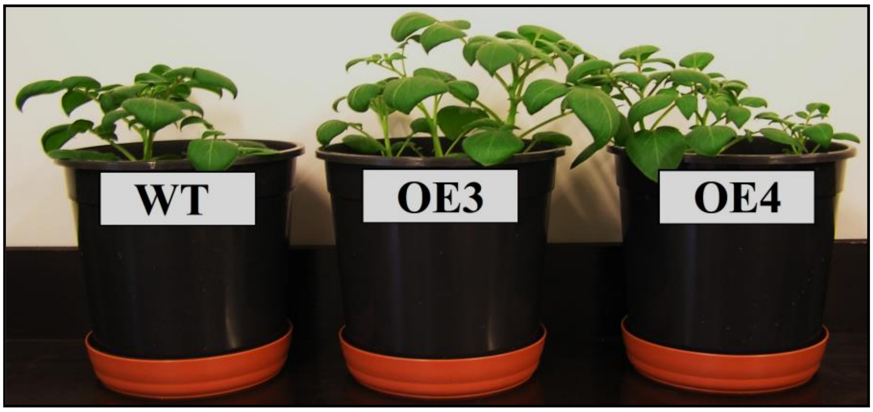

(B)

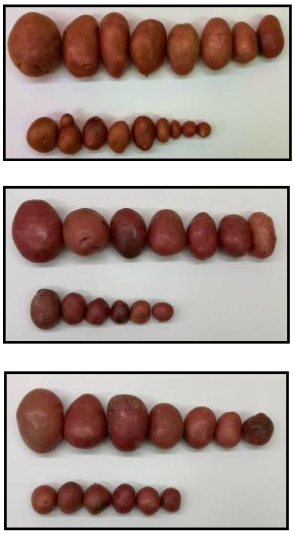

WT

***StBBX24*-OE3**

***StBBX24*-OE4**

Figure S5. Phenotype of *S. tuberosum* lines overexpressing *StBBX24*.

(A) Phenotype of WT and *StBBX24*-OE3 and *StBBX24*-OE4 transgenic plants grown in standard conditions (20°C±1, 40 % relative humidity and PFD of 350 µmoles photons m^{-2} s^{-1}) under a 14-h photoperiod for 3 weeks. WT - wild type.

(B) Tubers yield in WT, *StBBX24*-OE3 and *StBBX24*-OE4 transgenic plants grown in the growth room in standard conditions (20°C±1, 40 % relative humidity and PFD of 350 µmoles photons m^{-2} s^{-1}) under a 16-h photoperiod for 3 months. WT - wild type.
